# KRAB-Induced Heterochromatin Effectively Silences *PLOD2* Gene Expression in Somatic Cells and Is Resilient to TGFβ1 Activation

**DOI:** 10.3390/ijms21103634

**Published:** 2020-05-21

**Authors:** Rutger A. F. Gjaltema, Désirée Goubert, Christian Huisman, Consuelo del Pilar García Tobilla, Mihály Koncz, Pytrick G. Jellema, Dandan Wu, Uilke Brouwer, Antal Kiss, Pernette J. Verschure, Ruud A. Bank, Marianne G. Rots

**Affiliations:** 1 Epigenetic Editing Laboratory, Department of Pathology and Medical Biology, University of Groningen, University Medical Center Groningen, Hanzeplein 1 EA11, 9713 GZ Groningen, The Netherlands; gjaltema@molgen.mpg.de (R.A.F.G.); d.goubert@umcg.nl (D.G.); kriztian.huisman@gmail.com (C.H.); conpily@gmail.com (C.d.P.G.T.); p.g.jellema@umcg.nl (P.G.J.); wwwgrass123@163.com (D.W.); u.brouwer@umcg.nl (U.B.); 2 MATRIX Research Group, Department of Pathology and Medical Biology, University of Groningen, University Medical Center Groningen, 9713 GZ Groningen, The Netherlands; r.a.bank@umcg.nl; 3 Institute of Biochemistry, Biological Research Centre, H-6726 Szeged, Hungary; konczmisa@gmail.com (M.K.); kiss.antal@brc.hu (A.K.); 4 Doctoral School of Biology, Faculty of Science and Informatics, University of Szeged, H-6726 Szeged, Hungary; 5 Swammerdam Institute for Life Sciences, University of Amsterdam, Science Park 904, 1098 XH Amsterdam, The Netherlands; P.J.Verschure@uva.nl

**Keywords:** epigenetic editing, KRAB, gene repression, *PLOD2*, fibrosis, cancer

## Abstract

Epigenetic editing, an emerging technique used for the modulation of gene expression in mammalian cells, is a promising strategy to correct disease-related gene expression. Although epigenetic reprogramming results in sustained transcriptional modulation in several in vivo models, further studies are needed to develop this approach into a straightforward technology for effective and specific interventions. Important goals of current research efforts are understanding the context-dependency of successful epigenetic editing and finding the most effective epigenetic effector(s) for specific tasks. Here we tested whether the fibrosis- and cancer-associated *PLOD2* gene can be repressed by the DNA methyltransferase M.SssI, or by the non-catalytic Krüppel associated box (KRAB) repressor directed to the *PLOD2* promoter via zinc finger- or CRISPR-dCas9-mediated targeting. M.SssI fusions induced de novo DNA methylation, changed histone modifications in a context-dependent manner, and led to 50%–70% reduction in *PLOD2* expression in fibrotic fibroblasts and in MDA-MB-231 cancer cells. Targeting KRAB to *PLOD2* resulted in the deposition of repressive histone modifications without DNA methylation and in almost complete *PLOD2* silencing. Interestingly, both long-term TGFβ1-induced, as well as unstimulated *PLOD2* expression, was completely repressed by KRAB, while M.SssI only prevented the TGFβ1-induced *PLOD2* expression. Targeting transiently expressed dCas9-KRAB resulted in sustained *PLOD2* repression in HEK293T and MCF-7 cells. Together, these findings point to KRAB outperforming DNA methylation as a small potent targeting epigenetic effector for silencing TGFβ1-induced and uninduced *PLOD2* expression.

## 1. Introduction

Misregulation of epigenetic modifications is associated with aberrant gene expression profiles, which contribute to the development or progression of a wide spectrum of diseases [1]. The enzymes that catalyze epigenetic modifications have therefore been subjected to the intensive investigation as potential targets for treatment [2]. However, as inhibitors of epigenetic enzymes can result in genome-wide changes in chromatin and non-chromatin targets, these epi-drugs have undesired side-effects. Therefore, gene-targeting approaches designed to locally interfere with transcriptional activity provide promising alternatives to enzyme inhibitor strategies. 

**Figure 1:**
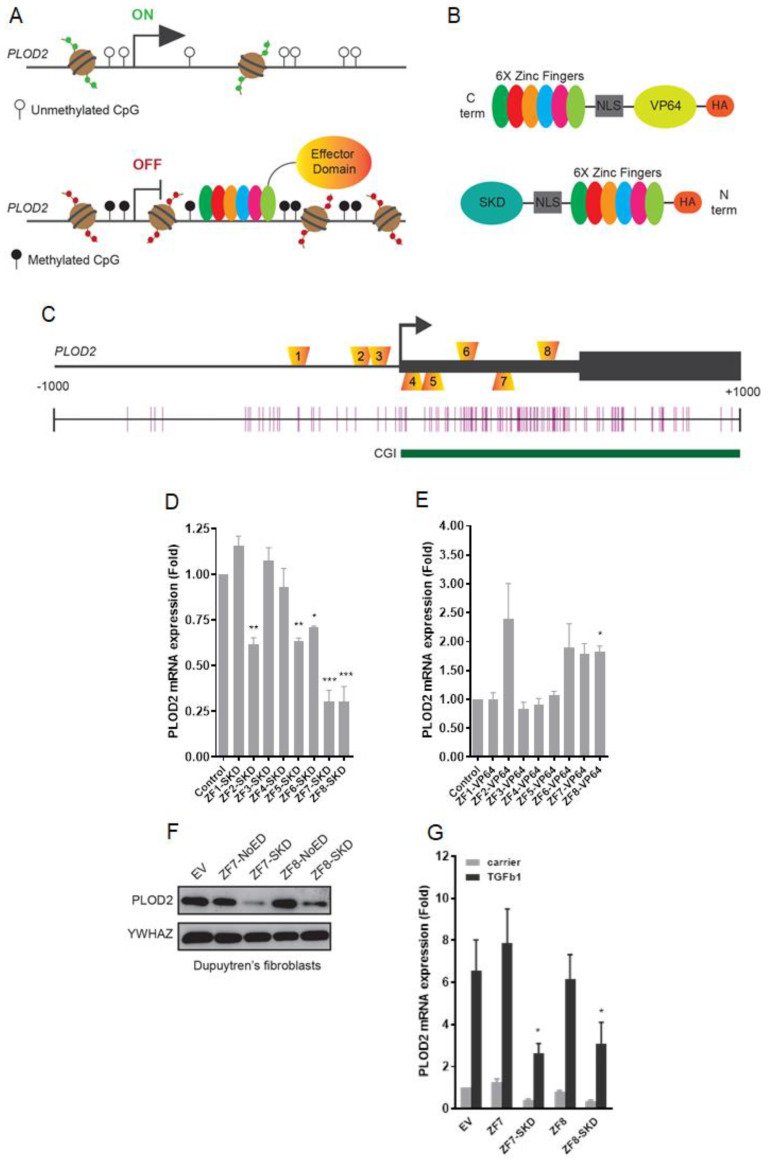
Screening of engineered transcriptional activators and repressors to target *PLOD2*. (**A**) Schematic representation of the epigenetic editing strategy to modulate *PLOD2* expression; (**B**) Schematic representation of the six-finger zinc finger (ZF) DNA binding domain with the fused effector domain Super Krüppel associated box (KRAB) Domain (SKD) or VP64 flanked by a nuclear translocation signal (NLS); (**C**) Approximate locations of the 8 ZFs binding sites in the *PLOD2* gene ranging from the proximal promoter to the first exon on both the leading and lagging strand. In the panel beneath, the CG island CG island (CpG) sites are depicted as vertical bars and a CpG island (CGI) as a green horizontal bar. (**D**) *PLOD2* mRNA expression levels in human dermal fibroblasts (HDFs) transduced with retrovirus to express the eight ZF-SKD fusion proteins, or with empty vector (EV) control (mean ± SEM; *n* = 3, one-way ANOVA (* *p* < 0.05, ** *p* < 0.01, *** *p* < 0.001). (**E**) *PLOD2* mRNA expression levels of HDFs transduced with retrovirus for the eight ZF-VP64 fusion proteins or EV control (mean ± SEM; *n* = 3, one-way ANOVA (* *p* < 0.05). (**F**) Western blot of Dupuytren’s patient-derived fibroblasts after retroviral transduction of ZF7-NoED, ZF7-SKD, ZF8-NoED, ZF8-SKD or EV control, stained for *PLOD2* and *YWHAZ* as a loading control. (**G**) *PLOD2* mRNA expression levels in HDFs after retroviral expression of ZFs or EV control and stimulated with TGFβ1 for 2 days (mean ± SEM; *n* = 3, one-way ANOVA (* *p* < 0.05).

Several designer DNA binding platforms are currently applied for gene targeting, e.g., Zinc Fingers Nucleases, TALENs, and CRISPR-Cas9 [3]. These platforms have been successfully repurposed as targetable transcriptional modifiers by removing the nuclease domain/activity and tethering them to transcriptional effectors or epigenetic enzymes. Targeted overwriting of epigenetic marks, referred to as epigenetic editing (Figure 1A,B) [4], has been successfully applied in diverse therapeutic models [5,6], however mitotic stability of the induced effects is still largely unclear. 

Generally, therapeutic effects have been achieved by exploiting episomal (AAV) or integrative (lentiviral) gene therapy vectors, which are increasingly accepted for gene editing in clinical trials [7], but which do not allow to investigate the mitotic stability of the induced epigenetic effects. Using Krüppel associated box (KRAB) as an effector domain, Thakore et al., showed silencing of *Pcsk9*, a regulator of cholesterol levels, in the liver of adult mice for a duration of at least 24 weeks upon delivery using AAV vectors [5]. However, as these delivery vectors are episomally maintained, it was not possible to assess the mitotic stability of the effect of KRAB itself. The mode of action of KRAB is to recruit KRAB-associated protein 1 (KAP-1), which in turn attracts H3K9me3 methyltransferase SETDB1 (attracting Heterochromatin Protein 1 (HP1)) and the NuRD complex to deacetylate histones and subsequently impact H3K4me3 levels [8]. Despite these indirect effects on the chromatin, targeting KRAB generally represses gene expression in a transient manner in somatic cells [4,9,10,11,12,13,14]. For the induction of stable heterochromatin, direct editing of epigenetic marks e.g., DNA methylation was assumed to be more effective [15,16].

There are contradictory reports on the sustainability of the changes induced by epigenetic editing: For transcriptional activation, e.g., one study reported that the stability of the transcription induced by exogenous writing of H3K4me3 histone modification is dependent on the endogenous DNA methylation state [17]. Similarly, sustained repression was achieved using targeted DNA methylation in some studies [10,15,18,19], but this could not be confirmed for other target genes [20,21]. These examples show that the maintenance of epigenetic reprogramming is context-dependent. 

The current consensus holds that for sustained effects of epigenetic reprogramming multiple effector domains are required [12,13,14,22]. For clinical applications however, the requirement of multiple components would be a serious limitation. Here, we tested two effector domains for their capability to induce direct and indirect epigenetic modifications, (long-term) gene repression and the effect of transcriptional activation on the induced heterochromatin. 

We focused on a clinically important gene, Procollagen-Lysine, 2-Oxoglutarate 5-Dioxygenase 2 (*PLOD2*), which has a progressive and metastasizing function in cancer [23]. *PLOD2* is also an important player in fibrosis, where it is induced by transforming growth factor beta-1 (TGFβ1) [24,25,26]. *PLOD2*, also known as lysyl hydroxylase 2 (*LH2*), is a collagen biosynthesis enzyme that initiates pyridinoline cross-links of collagens [27]. These cross-links prevent collagen degradation by proteinases and in turn force a feedback loop that results in excessive accumulation of collagen and disease progression [24,28]. Attenuating *PLOD2* expression has previously been shown to be a promising treatment against fibrosis and cancer metastasis in preclinical settings [29,30,31]. However, current approaches are either not selective for the *PLOD2* gene or are exploiting methods that are clinically less favorable (e.g., gene knockout) [28]. To induce repression of genomic *PLOD2*, we targeted the transcription factor KRAB and variants of the CG-specific prokaryotic DNA methyltransferase M.SssI to the *PLOD2* promoter region. Our results show that the M.SssI-induced DNA methylation did not affect endogenous *PLOD2* expression, but severely hampered the TGFβ1-induced activation of the gene. Interestingly, the expression of *PLOD2* was completely repressed by targeting of the transcriptional repressor KRAB to the *PLOD2* gene, even under conditions of continuous stimulation by TGFβ1.

## 2. Results

### 2.1. Engineered Transcription Factors Can Activate and Repress PLOD2 Expression

Eight modular six-finger zinc finger proteins (ZF1-ZF8) (Supplementary Figure S1) were engineered to bind 18 bp sequences in the genomic locus of *PLOD2* (Supplementary Figure S2), spanning a region from −150 to +479 bp relative to the transcription start site (TSS) (Figure 1C, Supplementary Figure S2B). To determine the efficiency of the ZF modules, we first expressed the eight ZFs fused to a variant of the KRAB suppressor (Super KRAB Domain (SKD)) or the transcriptional activator VP64 (tetramer of the Viral Protein VP16) (Figure 1B) in human dermal fibroblasts (HDFs). *PLOD2* expression levels were assessed 48 h after retroviral delivery. *PLOD2* mRNA expression was repressed by fusions of SKD to ZF2, ZF5, ZF6, ZF7, and ZF8 with ZF7 and ZF8 showing the strongest repression (70%, Figure 1D). For VP64 fusions, the strongest effects were observed with ZF2 -ZF6, -ZF7 and -ZF8 (Figure 1E). For ZF1, -ZF3 and -ZF4 no effect was observed for either fusion. No clear correlation was found between the expression of the respective ZF and the effect on *PLOD2* mRNA expression modulation (Supplementary Figure S3A). Based on this screening and their high protein expression levels (Supplementary Figure S3C), we continued our studies with ZF7 and ZF8.

### 2.2. ZF Repressors Attenuate Fibrosis-Related PLOD2 Expression

As our aim was to repress *PLOD2* in fibrotic fibroblasts, we assessed the effects of ZF-SKD fusions in patient-derived fibroblasts. We obtained fibrotic fibroblasts from a patient with Dupytren’s disease, a disabling fibrotic condition of the hand, where the palmar fascia is chronically affected by fibrotic tissue, and which results in a contracting phenotype restricting the motion of one or more fingers. Fibroblasts isolated from the affected palmar fascia were transduced to express ZF-SKD fusions. In this disease model, *PLOD2* protein production was strongly repressed at day-two post-infection when compared to the ZF without effector domain (NoED) and the empty vector (EV) controls (Figure 1F).

Using our healthy HDF model, we could simulate the fibrosis-related increase in *PLOD2* expression by TGFβ1. In the continuous presence of TGFβ1, both ZF-SKD fusions significantly repressed TGFβ1-induced *PLOD2* mRNA expression by 65%, five days post retroviral infection, whereas no effects were seen for the NoED and EV controls (Figure 1G). As TGFβ1 stimulations had no negative effects on ZF expression (Supplementary Figure S3B), this in vitro fibrosis model was used in further experiments. 

### 2.3. Both ZF-SKD as Well as ZF-M.SssI Induce Efficient Repression of PLOD2 in Fibroblasts

**Figure 2:**
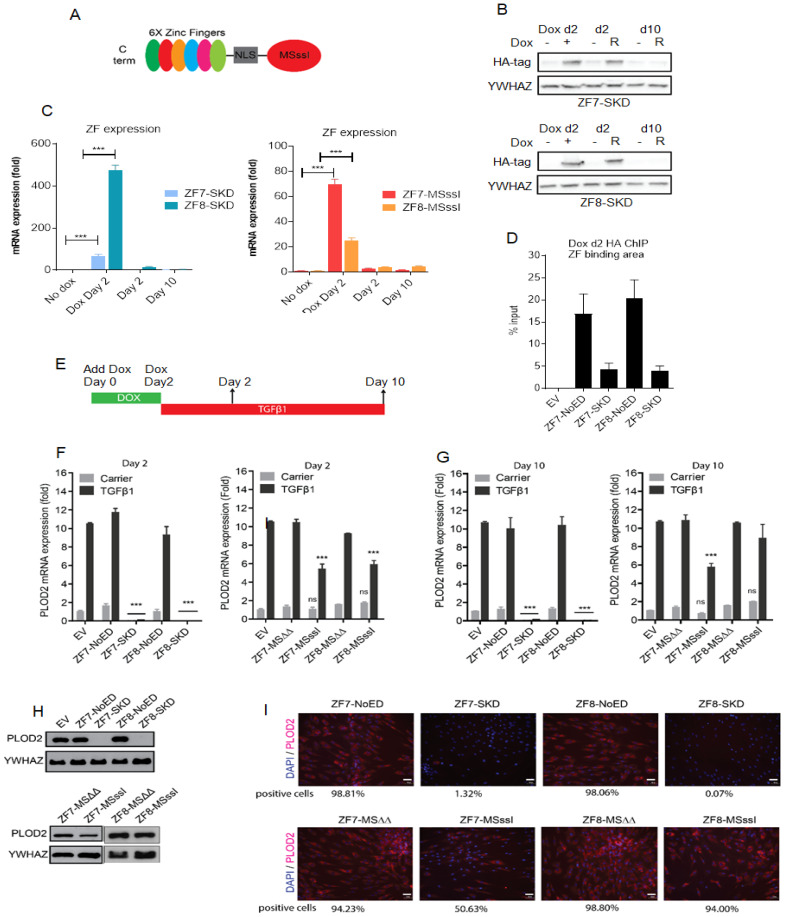
Zinc finger mediated targeting of SKD or M.SssI to the *PLOD2* promoter. (**A**) Schematic representation of the DNA methylation editors containing M.SssI (or derivates) C-terminally fused to 6-finger ZF with NLS in between. (**B**) mRNA expression levels of ZF-SKD or ZF-M.SssI fusions in HDFs engineered to express ZF-fusions treated with doxycycline for two days (Dox Day2) and stimulated with TGFβ1 for additional 2 and 10 days (mean ± SEM; n = 3, unpaired Student’s *t*-test (*** *p* < 0.001)). (**C**) Western blot of transgenic HDFs after doxycycline treatment for two days (Dox Day 2), and subsequent TGFβ1 stimulation for 2 and 10 days (Day 2, Day 10), compared to non-dox treated cells (lanes labeled as “-”). Stained for His-tagged ZF7-SKD and ZF8-SKD and YWHAZ as a loading control. (**D**) qChIP assay with an antibody against HA-tag to reveal binding of the ZFs at the target region (+326/+447) directly following the 2 days of doxycycline treatment. (**E**) Scheme of the experimental procedure: transgenic HDFs were treated with doxycycline for 2 days (Dox Day 2), followed by 2 or 10 days of stimulation with TGFβ1 or vehicle control. (**F**) *PLOD2* mRNA expression levels of transgenic HDFs after doxycycline treatment and stimulation with TGFβ1 or control (PBS with BSA and citric acid) for 2 days (mean ± SEM; *n* = 3, one-way ANOVA (*** *p* < 0.001; ns = not significant)). (**G**) *PLOD2* mRNA expression levels of transgenic HDFs after doxycycline treatment and stimulation with TGFβ1 or control for 10 days (mean ± SEM; *n* = 3, one-way ANOVA (*** *p* < 0.001; ns = not significant))**.** Statistical differences were compared to EV stimulated with TGFβ1. (**H**) Western blot of transgenic HDFs treated with doxycycline and stimulated with TGFβ1 for 2 days, stained for PLOD2 and YWHAZ as a loading control. (**I**) PLOD2 protein expression in transgenic HDFs treated with doxycycline and subsequently stimulated with TGFβ1 for 10 days, determined by immunocytochemistry and quantified by TissueFAXS relative to DAPI as a percentage of positive cells. Depicted white bars represent 20 µm.

To ensure the expression of the tested epigenetic effectors in all analyzed cells, we created HDF cell lines carrying stably integrated TET-ON doxycycline responsive transgene cassettes encoding one of the ZF-SKD, ZF-M.SssI and ZF-M.SssIΔΔ fusion proteins (Figure 2A). M.SssI is a CG-specific prokaryotic DNA (cytosine-5) methyltransferase and M.SssIΔΔ is an inactive double mutant (Y137F+C141S) of M.SssI. M.SssIΔΔ was used as the negative control. Treatment of the cells with doxycycline for two days resulted in increased ZF-SKD and ZF-M.SssI mRNA expression (Figure 2B). Two days after doxycycline withdrawal, expression of the fusion genes dropped back to pre-induction levels (e.g., cycle thresholds for ZF7-SKD expression range from Ct = 25 before Dox, to Ct = 19 after two days of Dox treatment (Dox day 2), back to Ct = 24 after two days subculturing (Day 2); compared to YWHAZ which yields Ct values of 20 in these qRT-PCR experiments). Efficient protein expression of the ZF-SKD fusions was detected two days after doxycycline withdrawal (Figure 2C), whilst the expression was no longer detectable after 10 days. The HA-tagged ZF7 and ZF8 NoED or fused to SKD were highly enriched at the targeted DNA region of *PLOD2* confirming efficient binding to the addressed sites (Figure 2D).

The effects of ZF-SKD and ZF-M.SssI expression was studied under two conditions: in transgenic HDF cells in which *PLOD2* expression was induced with the transcriptional activator TGFβ1, and in transgenic HDF cells, which were not treated with TGFβ1 (carrier). Expression of the ZF-SKD fusions resulted in almost complete prevention of TGFβ1-induced as well as unstimulated *PLOD2* mRNA expression, and this strong repression was detectable even after 10 days of continuous TGFβ1-stimulation (Figure 2F,G). In contrast, the expression of the ZF-M.SssI fusions prevented TGFβ1-induced *PLOD2* mRNA levels only by 50% on day 2 (Figure 2F), and this effect was present at day 10 for ZF7-M.SssI, but not for ZF8-M.SssI (Figure 2G). As expected, NoED controls or the catalytically inactive MSΔΔ had no effect on the TGFβ1-induced *PLOD2* mRNA expression. Interestingly, non-stimulated *PLOD2* expression (carrier control without TGFβ1 stimulation) was repressed only by SKD fusions, which suggests that targeted DNA methylation can be used to repress fibrosis-specific overexpression of *PLOD2*, leaving the physiological expression levels unaltered. Importantly, neither TGFβ1-stimulation alone (Supplementary Figure S4A), nor in combination with ZF-SKD expression (Figure 2I) had adverse effects on cell morphology. 

Changes detected at the mRNA level were reflected at the protein level. After two days of Dox treatment and two additional days of continuous TGFβ1 stimulation, no PLOD2 protein was detectable by Western blot in cells engineered to contain the ZF-SKD transgene, whereas a substantial reduction in PLOD2 protein was observed for cells engineered to contain the ZF-M.SssI transgene (Figure 2H). Importantly, also after 10 days of TGFβ1 stimulation, immunocytochemistry and quantification of positive cells confirmed that PLOD2 was repressed (by ~99% for both ZF-SKD and 50% for ZF7-M.SssI (Figure 2I, Supplementary Figure S4B)). Collectively, the mRNA, as well as the protein levels, showed effective *PLOD2* repression, even after 10 days of continuous expression stimulation, for both ZF-SKDs as well as for ZF7-M.SssI in our fibrosis model.

### 2.4. PLOD2 Repression is Associated with Epigenetic Modulation in TGFβ1 Stimulated Fibroblasts 

**Figure 3:**
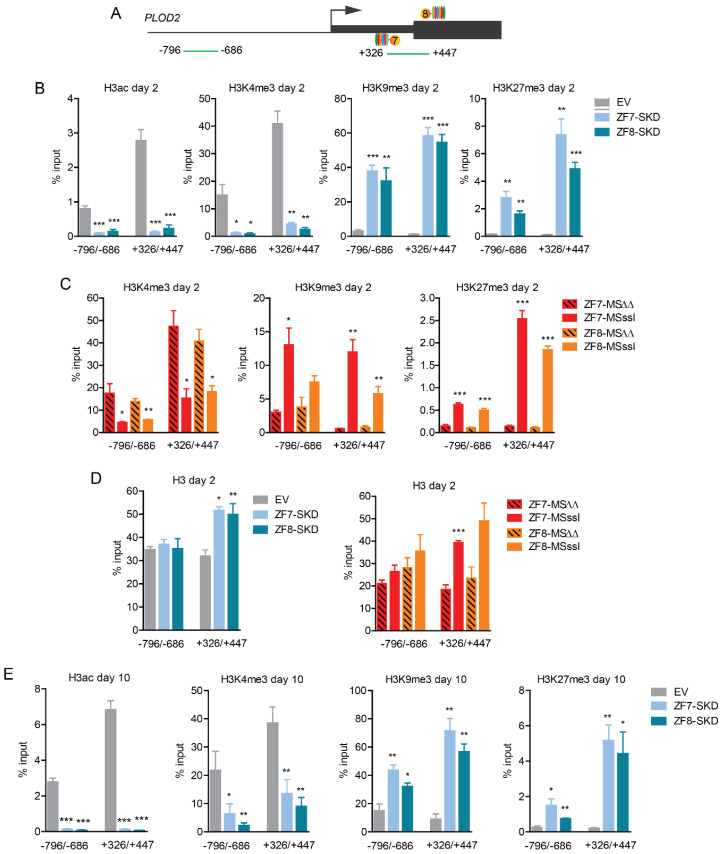
Induced *PLOD2* repression is accompanied by repressive histone modifications in human dermal fibroblasts stably engineered to express the indicated ZF-fusions. (**A**) Schematic representation of the *PLOD2* target regions detected by qPCR (+326 to +447 and −796 to −686)) upon chromatin pull-down using the indicated antibodies (qChIP). (**B**) qChIP assay with antibodies against H3ac, H3K4me3, H3K9me3 and H3K27me3 of the indicated transgenic cells after 2 days of stimulation with TGFβ1. (**C**) qChIP assay with antibodies against H3K4me3, H3K9me3, H3K27me3 of the indicated transgenic cells after 2 days of stimulation with TGFβ1. (**D**) qChIP assay with antibodies against histone 3 (H3) of the indicated transgenic cells after 2 days of stimulation with TGFβ1. (**E**) qChIP assay with antibodies against H3ac, H3K4me3, H3K9me3 and H3K27me3 of the indicated transgenic cells after 10 days of stimulation with TGFβ1. Data are shown as mean ± SEM; n = 3, unpaired two-tailed Student’s *t*-test (* *p* < 0.05, ** *p* < 0.01, *** *p* < 0.001).

To identify the epigenetic changes that underlie the observed *PLOD2* repression, we first analyzed two regions of the *PLOD2* gene for histone modifications in doxycycline-treated HDFs after two days of TGFβ1 stimulation (Figure 3A). One of the investigated regions (from +326 to +447 bp relative to TSS) contained the target sites of ZF7 and ZF8, whereas the other region (from −796 to −686 bp) was located ~1kb upstream. For both SKD fusions, a reduction of the gene activation-related modifications H3ac and H3K4me3, and an increase of the repression-related modifications H3K9me3 and H3K27me3 were observed at the target region (from +326 to +447), as well as at the −796 to −686 bp upstream region (Figure 3B). Targeting M.SssI by the zinc fingers ZF7 and ZF8 also reduced H3K4me3, and increased H3K27me3 and H3K9me3 levels at both regions compared to cells targeted with the catalytically-inactive M.SssIΔΔ (Figure 3C). 

Furthermore, both SKD and M.SssI targeted cells showed enrichment of Histone 3 (Figure 3D), suggesting enhanced nucleosome occupancy predominantly at the target site. To test the resilience of the epigenetic modulations indirectly induced by SKD, we analyzed activating and repressive histone marks in doxycycline-treated cells after 10 days of continuous TGFβ1 stimulation of *PLOD2* expression. We again observed a significant reduction of H3ac and H3K4me3, and significant enrichment of H3K9me3 and H3K27me3 compared to EV at both regions (from −796 to −686 and from +326 to +447) (Figure 3E), even though the cells had a severely reduced proliferation rate during serum starvation (Supplementary Figure S5). These data show that the repressive chromatin environment induced by targeting ZF-SKD fusions is resilient to long-term stimulation with TGFβ1.

**Figure 4:**
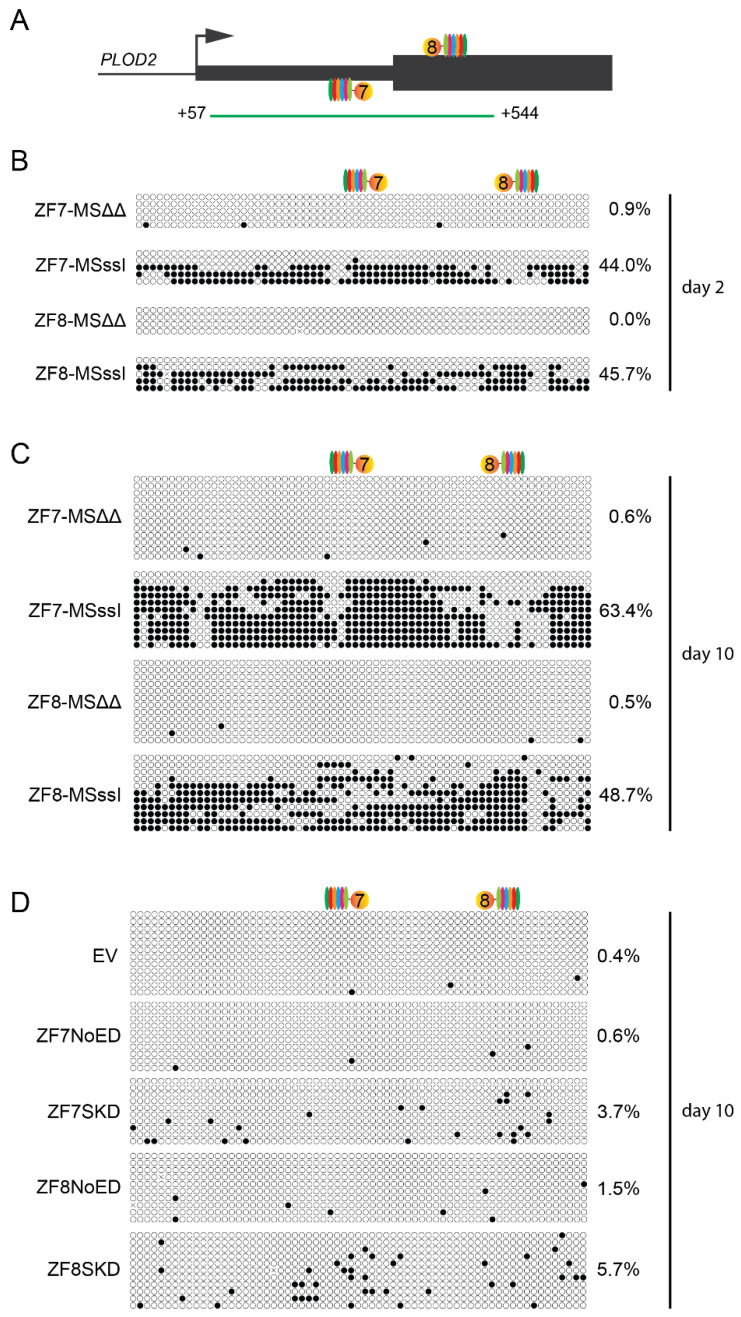
Analysis of DNA methylation by bisulfite sequencing in a segment of the *PLOD2* gene in human dermal fibroblasts. Open circles represent unmethylated CpGs, closed circles represent methylated CpGs. The total percentage of DNA methylation is shown on the right side of the panels. (**A**) Schematic representation of the region probed for methylation (from +57 to +544 relative to the transcriptional start site). The numbers 7 and 8 indicate approximate positions of the target sites for the zinc fingers ZF7 and ZF8, respectively. (**B**) Cells stably engineered to contain the ZF-M.SssI transgene. Methylation was determined after two days of Dox treatment followed by two days of TGFβ1 stimulation. (**C**) The same as B, but methylation was determined after ten days of TGFβ1 stimulation. (**D**) Cells stably engineered to contain the ZF-SKD transgene. Methylation was determined after two days of Dox treatment followed by ten days of TGFβ1 stimulation.

Bisulfite sequencing of the +57 to +544 bp region of the *PLOD2* gene (Figure 4A) detected efficient de novo DNA methylation in cells expressing ZF7-M.SssI or ZF8-M.SssI after two days of TGFβ1 stimulation (Figure 4B). For ZF7-M.SssI the level of DNA methylation increased even further after ten days of stimulation (Figure 4C). Extensive methylation was detected throughout the analyzed region without a clear methylation peak adjacent to the targeting sites. Interestingly, ZF8-M.SssI expressing cells showed similar DNA methylation levels at day 2 and day 10, which contrast with the loss of transcriptional repression by day 10 (Figure 2G,I). Despite the fact that targeting SKD resulted in very effective *PLOD2* repression, only low levels of DNA methylation were induced (3.7% for ZF7-SKD and 5.7% for ZF8-SKD compared to 0.4% for EV and up to 1.5% for NoED (Figure 4D)). Taken together, an increase in DNA methylation could be observed for both ZF7-M.SssI and ZF8-M.SssI, and not for the ZF-SKD fusions, confirming that the M.SssI and SKD effector domains induce *PLOD2* repression via different regulatory mechanisms.

### 2.5. SKD- and M.SssI-Induced Epigenetic Modulation in Highly Proliferative Breast Cancer Cells

**Figure 5:**
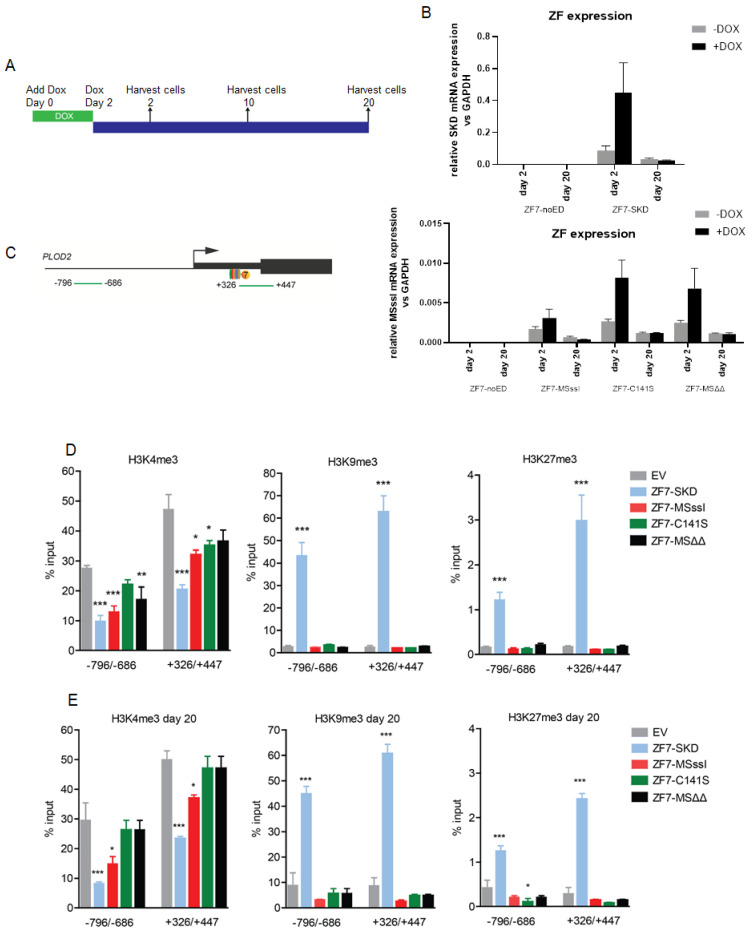
Targeting SKD, but not M.SssI, introduces repressive histone modifications in transgenic MDA-MB-231 cells. Expression of ZF-fusions in MDA-MB231 cells stably engineered to contain the indicated transgenes, after treatment with doxycycline for 2 days and subcultured for an additional 2 and 20 days. (**A**) Schematic representation of the experimental procedures for TET-ON MDA-MB-231 transgenic cells; (**B**) mRNA expression levels of ZF-SKD or ZF-M.SssI fusions in transgenic MDA-MB-231 cells treated with or without doxycycline for two days followed by 2 or 20 days subculturing. The specificity of the primers is indicated by the absence of expression in ZF7-NoED cells. (**C**) Schematic representation of *PLOD2* with the ZF7 target site and areas detected by qChIP. (**D**) qChIP assay on transgenic cells subcultured for 2 days using antibodies against H3K4me3, H3K9me3 and H3K27me3, represented as enrichment against input DNA (mean ± SEM; n = 3, one-way ANOVA * *p* < 0.05, ** *p* < 0.01, *** *p* < 0.001). (**E**) qChIP assay on transgenic cells subcultured for 20 days using antibodies against H3K4me3, H3K9me3 and H3K27me3, represented as enrichment against input DNA (mean ± SEM; n = 3, one-way ANOVA (* *p* < 0.05, ** *p* < 0.01, *** *p* < 0.001)).

To further validate the epigenetic modulation potential of SKD and M.SssI, we stably integrated the ZF-SKD and ZF-M.SssI fusion genes into the genome of highly proliferating MDA-MB-231 breast cancer cells, in which high *PLOD2* expression was shown to be associated with metastatic potential [30]. To reduce potential off-target DNA methylation induced by WT M.SssI, we included the C141S mutant of M.SssI, which has ~1% activity of the wild type enzyme [32,33,34]. In these experiments, ZF7 was used as a targeting domain since this was the most efficient DNA binding domain in our HDF system. After doxycycline treatment for two days, the cells were subcultured for an additional 2 and 20 days in regular medium (Figure 5A), at which time points the cells were assessed for any remaining expression of ZF-fusions (Figure 5B), and for epigenetic changes at two regions (Figure 5C). 

We first tested epigenetic changes by chromatin immunoprecipitation. H3K4me3 levels at the ZF7 binding region (from +326 to +447) were reduced by ~58% in ZF7-SKD expressing cells, and to a lesser extent by the different ZF7-M.SssI derivatives at day 2 (Figure 5D). A similar reduction of H3K4me3 levels for both ZF7-SKD and ZF7-M.SssI compared to EV was detected in the −796 to −686 region. Again, ZF-SKD expressing cells showed strong enrichment in H3K9me3 and in H3K27me3 at both investigated regions as compared to EV expressing cells (Figure 5D). In contrast to our observations in HDFs (Figure 3C), targeting of M.SssI WT to the *PLOD2* region in MDA-MB-231 cells did not result in secondary changes of H3K9me3 and H3K27me3 levels two days after doxycycline removal. This could be due to differences in growth kinetics, which entails a much faster turnover of MDA-MB-231 cells, or to the continuous TGFß1 stimulation applied to the HDFs but not to the MDA-MB-231 cells. 

After 20 days of subculturing the cells in a regular medium, we found reduced levels of the activating signal H3K4me3 in both analyzed regions of the SKD- and M.SssI-targeted cells, but not in cells expressing ZF7-C141S or ZF7-MSΔΔ (Figure 5E). In SKD-targeted cells, clear enrichments of the repressive marks H3K9me3 and H3K27me3 were seen. In contrast, ZF-M.SssI targeted cells showed no enrichment of these histone modifications (Figure 5D,E). 

**Figure 6:**
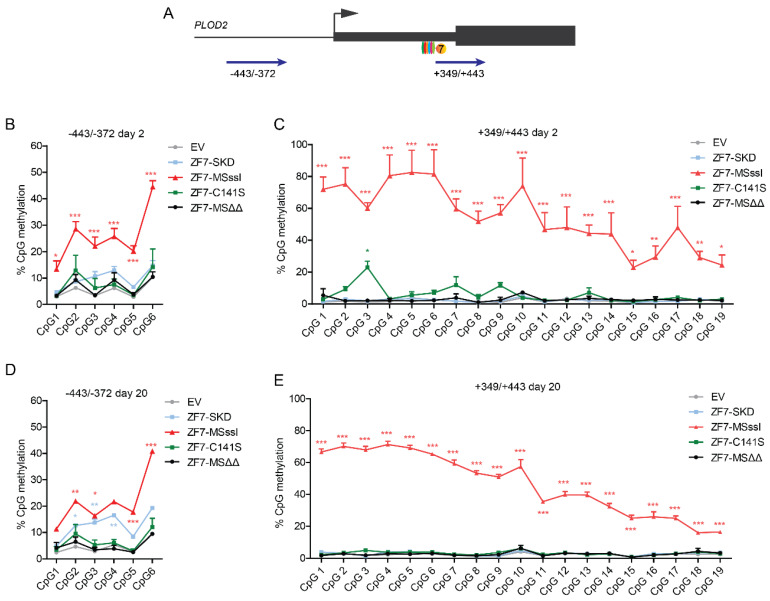
Targeting M.SssI induces strong and long-range DNA methylation in transgenic MDA-MB-231 cells. MDA-MB-231 cells, stably engineered to contain the indicated transgenes, were treated with doxycycline for 2 days and subcultured for an additional 2 and 20 days. (**A**) Schematic representation of the *PLOD2* locus. The approximate locations of the ZF7 binding site, the transcriptional start size, and the two regions (−443 to −372 and +349 to +443) analyzed for induced DNA methylation are indicated. (**B**–**E**) DNA CpG methylation levels quantified by pyrosequencing (**B**) region −443 to −372, two days after doxycycline withdrawal; (**C**) region +349 to +443, two days after doxycycline withdrawal; (**D**) region −443 to −372, 20 days after doxycycline withdrawal; (**E**) region +349 to +443, 20 days after doxycycline withdrawal. All pyrosequencing data are depicted as mean ± SEM; n = 3, two-way ANOVA * *p* < 0.05, ** *p* < 0.01, *** *p* < 0.001.

Next, DNA methylation changes were analyzed in two regions (from −443 to −372 and from +349 to +443) of the *PLOD2* gene (Figure 6A). After two days of subculturing the cells in regular medium, an increase of DNA methylation by M.SssI was observed at all assessed CpGs upstream of the *PLOD2* transcription start site (Figure 6B) and within the ZF targeting region (Figure 6C). Based on the absolute levels, the de novo DNA methylation was the highest around the targeting site and faded towards both extremities. Interestingly, targeting C141S induced significant methylation of one specific CpG site at the ZF target region of *PLOD2* (Figure 6C). SKD and the catalytically inactive MSΔΔ did not significantly affect DNA methylation in these regions. DNA methylation in both *PLOD2* regions of the M.SssI targeted cells were present after 20 days of subculturing (Figure 6D,E), while the other targeted M.SssI derivates did not affect DNA methylation at the *PLOD2* promoter at day 20. Taken together, these data indicate that M.SssI induce strong DNA methylation without repressive histone modification cross-talk in MDA-MB-231 breast cancer cells, whereas SKD induced pronounced heterochromatin features without DNA methylation.

### 2.6. ZF-SKD and ZF-M.SssI-Induced Repression of PLOD2 in Highly Proliferative Breast Cancer Cells

**Figure 7:**
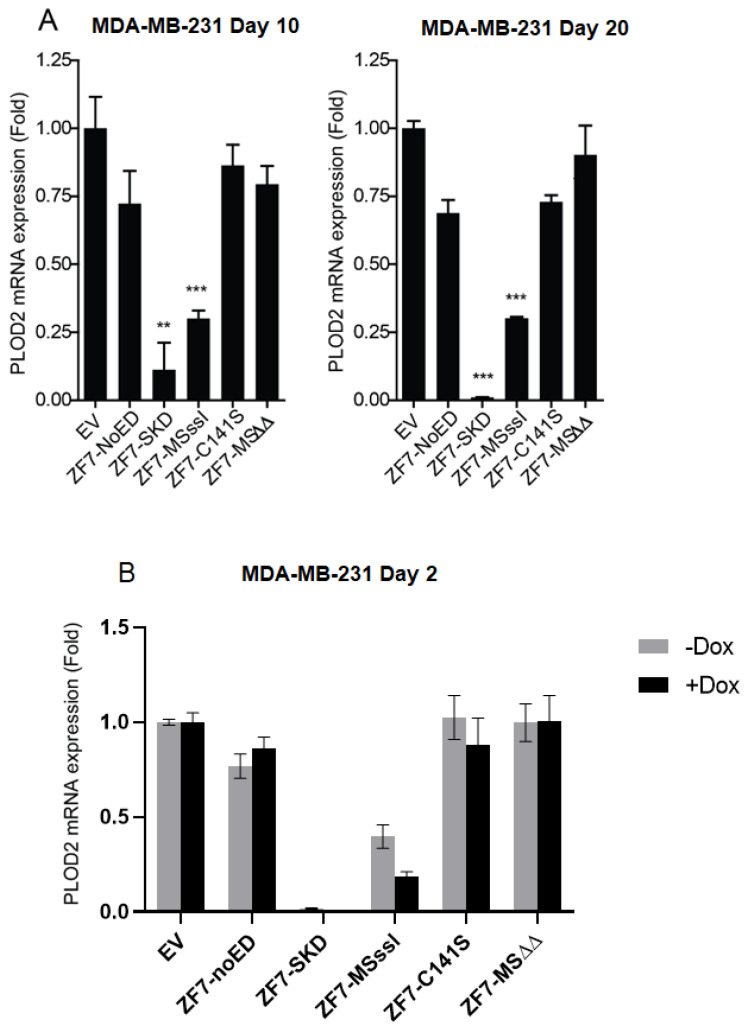
Repression of *PLOD2* by targeting SKD or M.SssI to the *PLOD2* promoter in transgenic MDA-MB-231 cells. (**A**) *PLOD2* mRNA expression levels in MDA-MB-231 cells, engineered to contain the indicated transgenes, treated with doxycycline for 2 days and subcultured in normal medium for 10 and 20 days (mean ± SEM; n = 3, one-way ANOVA ** *p* < 0.01, *** *p* < 0.001). (**B**) *PLOD2* mRNA expression levels in the transgenic MDA-MB-231 cells treated with or without doxycycline for 2 days and subcultured in normal medium for 2 days.

Next, we were interested in the functional effect of the rewritten epigenetic marks in the MDA-MB-231 cells engineered to contain the ZF-fusion transgenes. For transgenic MDA-MB-231 cells, we observed that SKD targeting resulted in almost complete inhibition of *PLOD2* mRNA expression, after ten or twenty days of subculturing in regular medium, while M.SssI targeting reduced *PLOD2* mRNA expression by ~80%, compared to cells expressing EV (Figure 7A). The C141S and the MSΔΔ mutants of M.SssI did not affect *PLOD2* mRNA expression. Based on other reports using this system [15,35], these data seem to indicate that SKD and M.SssI-induced repression of *PLOD2* is sustained in these highly proliferative breast cancer cells. Yet, since leakiness is an often-observed phenomenon for the TET ON system, we measured the *PLOD2* expression also in cells that were not treated with doxycycline and found that the background levels of ZF-fusions (Figure 5B) seemed sufficient to induce similar levels of *PLOD2* repression as observed for Dox-supplemented conditions (Figure 7B). This observation of highly effective uninduced low-dose SKD-mediated *PLOD2* silencing was also observed for another breast cancer cell line at day 2 (MCF-7, Supplementary Figure S6) and for days 10 and 20, time points of the transgenic MDA-MB-231 cells (Supplementary Figure S7A). 

Given that other studies [9,12,15] clearly showed a transient nature of SKD-induced repression, we set out to explore leakiness of the expression of EDs in our system and its effects on *PLOD2* expression in more detail (Supplementary results and Supplementary Figures S8 and S9). In short, in our experimental setup, the TET ON stable cell line system does not allow conclusions on the sustainability of SKD- or M.SssI-induced effects on *PLOD2* expression as repression was observed also without Dox treatment in various conditions and at longer time points (up to day 45).

### 2.7. SKD and M.SssI-Induced Repression of PLOD2 Using the Transient CRISPR-dCas9 Platform

Sustainability of SKD- and M.SssI-induced *PLOD2* repression was studied using a transient CRISPR gRNA expression system with dCas9 fused to SKD or to variants of MSssI, either constitutively expressed or also transiently expressed after plasmid transfection. *PLOD2* mRNA levels were measured at day 2 and day 12. Day 12 was chosen for assessing sustainability because by day 12 the expression of sgRNAs has faded out (Supplementary Figure S10A). 

**Figure 8:**
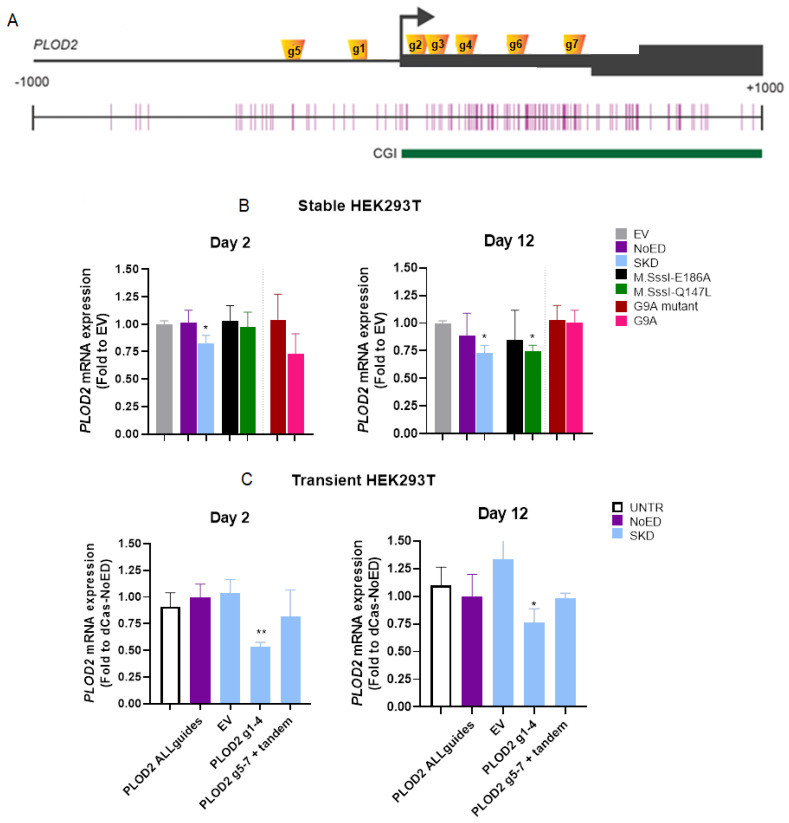
Sustained repression of *PLOD2* by SKD targeted to the *PLOD2* promoter by CRISPR-dCas9 in HEK293T cells. (**A**) Schematic representation of the *PLOD2* locus with binding locations of the sgRNAs (g1-7). (**B**) *PLOD2* mRNA expression levels in HEK293T cells constitutively expressing the indicated dCas-fusions, 2 and 12 days after transient transfection with sgRNA g1-4 plasmids. Data are shown relative to the *PLOD2* expression in the respective transgenic cells upon transfection with plasmids expressing “empty” guide RNA vector (EV), set at one for each stable cell line. (**C**) *PLOD2* mRNA expression levels of wild-type HEK293T cells, 2 and 12 days after transient transfection with plasmids encoding the indicated sgRNAs and dCas9-SKD (blue bars). Data are shown relative to PLOD2 expression in cells transfected with plasmids expressing all gRNAs and dCas9-NoED (No Effector Domain), set as 1 (purple bar). EV (empty vector) refers to sgRNA plasmids without the targeting nucleotides. Data are the mean ± SEM of biological triplicates, differences are analyzed using unpaired two-tailed Student’s *t*-test vs. EV (**B**) or vs. dCas9-NoED (**C**); * *p* < 0.05, ** *p* < 0.01.

First, transient transfections were performed to express sgRNAs (g1-4) targeting *PLOD2* in HEK293T cells, engineered to constitutively express dCas9 only (NoED) or dCas9 fused to SKD, to the inactive mutant of M.SssI (E186A), or to M.SssI-Q147L (which has ~10% activity of wild type M.SssI). The lower DNA binding affinity of Q147L has been proven to increase the specificity of targeted DNA methylation over the wildtype M.SssI [34]. To assess whether direct writing of H3K9me would result in sustained effects, as previously shown [36], HEK293T cells constitutively expressing dCas9 fused to the H3K9 methyltransferase G9A (or dCas9 fused to a catalytic inactive G9A (G9A mutant [37]) were used. In the HEK293T-dCas9-SKD cells, an initial repression of *PLOD2* was observed two days after the transfection of the PLOD sgRNAs, as compared to EV controls (Figure 8B). Of note, the induced repression is far less effective in the dCas9-SKD expressing cells compared to the ZF-SKD expressing cells as for cells expressing dCas9-SKD, the sgRNAs need to be delivered by transient expression plasmids. The repression was sustained and even further reinforced 12 days after transfection of sgRNAs (from 17.5% to 26.8%). In the M.SssI(Q147L) transgenic cell line initially no PLOD2 repression was observed upon transfection with sgRNA plasmids g1-4 when compared to the empty guide transfection control. After 12 days, however, a repression of 25.5% could be observed in cells transfected with PLOD2 guide plasmids compared to empty guide transfection control, which was not observed for M.SssI(E186A) (Figure 8B). Furthermore, the potential of dCas9-G9A to induce sustained *PLOD2* repression was also investigated in different cell types (HeLa and MCF-7, Supplementary Figure S10B), but no significant repression could be observed compared to the catalytically inactive dCas9-G9A mutant. Of note, for MCF-7 cells stably expressing G9A or its mutant, a 50% reduction in *PLOD2* expression was observed for PLOD2 g1-4 compared to empty guide control transfections (Supplementary Figure S10B). 

Next, we engineered stable constitutively dCas9-SKD expressing MCF-7 cells and transiently transfected plasmids expressing the *PLOD2* sgRNAs or the sgRNA empty vector. In these cells, SKD targeting of *PLOD2* led to sustained repression 14 days after *PLOD2* sgRNA transfection (Supplementary Figure S10C). Of importance, targeting another gene (*SPEDF*) in the same set of experiments did not lead to sustained repression, indicating that the *PLOD2* gene might be particularly sensitive to repression by KRAB-induced mechanisms and that KRAB effects are chromatin context-dependent. 

We also investigated whether transfecting other sgRNAs (PLOD2 g5-7) targeted to different positions in the *PLOD2* promoter (Figure 8A), or whether combining the four sgRNAs into one tandem plasmid (instead of using a mix of four separate plasmids) could improve the repression of *PLOD2* in HEK293T-dCas9-SKD cells. Only transfecting the combination of four separate plasmids expressing the guides PLOD2 g1-4 resulted in significant repression (Supplementary Figure S10D). 

Collectively, upon targeting *PLOD2* in engineered HEK293T cells, SKD led to sustained repression, while repression induced by M.SssI seems to take a longer time (detectable after 12 days).

Finally, to confirm the sustained character of the SKD-induced *PLOD2* repression, we transfected the plasmids to express dCas9-ED as well as the sgRNAs into wildtype HEK293T cells. Transient expression of dCas9-SKD and PLOD2 g1-4, resulted in sustained repression previously seen in stable dCas9-SKD-expressing cells. The SKD-induced repression was even more efficient in this system compared to the stable dCas9-SKD expressing cells with 46.3% repression after two days, compared to 17.5% repression in stable HEK293T-SKD. Importantly, also after transient transfection, *PLOD2* repression was sustained with 23.8% repression for at least 12 days (Figure 8C). Combining the PLOD2 tandem with PLOD2 g5-7, to cover a larger region within the *PLOD2* gene, did not lead to a significant downregulation of *PLOD2* (Figure 8C, Supplementary Figure S10D). In summary, using the truly transient CRISPR-dCas9 system, we showed that SKD is sufficient to induce sustained *PLOD2* repression. 

## 3. Discussion

In this study, we showed that targeting the KRAB domain (SKD) can lead to the silencing of the *PLOD2* gene without the induction of DNA methylation, even under continuous expression stimulation by TGFβ1. DNA methylation-induced by M.SssI did inhibit the TGFβ1-induced expression of *PLOD2* repression, while not affecting the constitutive *PLOD2* expression. By using the transient CRISPR-dCas9 platform we showed that targeting of SKD, or an M.SssI derivative, to *PLOD2* might be sufficient to achieve mitotically stable gene repression. As such, epigenetic editing using either of these effector domains has the potential to evolve into anti-PLOD2 therapeutics against fibrosis. Interestingly, targeted DNA methylation to inhibit *PLOD2* expression might even leave healthy cells unaffected. Yet, further research using these tools will be needed to unravel the differential clinical advantages. 

The current paradigm of KRAB induced repression holds that transcriptional effects are transient in somatic and cancer cells [4,9,10,11,12,13,22,38]. This transient nature of repression has been confirmed in transgenic mice using a drug-controllable KRAB repressor that targets the endogenous *Hprt* gene [39]. In contrast, multiple other studies showed a developmental-stage dependent effect of KRAB, where the expression of KRAB-repressors during early embryonic development induced stable repression, while expression at later stages of development resulted in reversible silencing [40,41,42,43]. Interestingly, in iPSCs, which are considered as embryonic models, KRAB-induced repression was fully reversible [44]. The stability of KRAB-induced gene repression in early stages of embryonic development was attributed to de novo DNA methylation, indirectly induced by KRAB [40,42,43]. When HP1 was targeted directly to *Oct4* or to reporter plasmids in embryonic stem cells, sustained DNA methylation was observed, which was associated with sustained repression in the absence of transcriptional activation [45,46]. Interestingly, also in these studies a clear context-dependency was observed regarding stable epigenetic reprogramming. De novo DNA methylation upon KRAB targeting was not detected in somatic cells [42,47]. Our study confirms the lack of DNA methylation in somatic cells upon targeting KRAB to *PLOD2*. This finding on longer-term KRAB-induced *PLOD2* epigenetic reprogramming in somatic cells is in contrast with studies showing that DNA methylation is required for maintenance of silencing [12,13,14,46,48]. 

Unraveling context-dependent effects is of importance as current understanding dictates that a combination of different repressive effector domains is needed for epigenetic editing to achieve sustained gene repression [12,13,14,22]. Although upon random integrations of GFP reporter cassettes, some genomic loci seemed responsive to KRAB for long-term silencing, Amabile et al. showed that Dnmt3A, Dnmt3L and KRAB together are superior over individual and two effector domain approaches in achieving long-term gene repression. Indeed, the three domains were required to ensure the repressive epigenetic state associated with long-term silencing of three endogenous genes (*B2M, IFNAR VEGFA*) [12]. Similarly, these EDs, when fused together in a single TALE-based fusion protein, led to robust and sustained silencing of *CXCR4* (although not of *CCR5*), accompanied by reduced chromatin accessibility and increased promoter methylation [14]. By using the CRISPR platform, O’Geen et al. showed that co-targeting of dCas9-KRAB and dCas9-Dnmt3A3L can induce long-term repression for some genes [13]. Importantly, these authors also presented examples of genes that could not be repressed by this combination. In this respect, a combination of Dnmt3A3L and EZH2 (but not KRAB) was later demonstrated to be required to stably repress *HER2*, while again Dnmt3A3L and/or KRAB were sufficient to induce long-term repression of *SNURF* [22]. To understand context-dependent epigenetic transcription regulation, which involves both stable sustained and dynamic flexible aspects, models to obtain a mechanistic understanding of its systems behavior are needed. Quantitative measurements of targeted epigenetic editing with read-outs at real-time, single-cell and single-molecule level combined with dynamic computational models are important to formulate and verify mechanistic models. Our current epigenetic editing study provides directions for such a quantitative model-driven approach.

Despite the yet unknown context-dependent requirements for gene silencing, epigenetic editing is considered to require more than KRAB to maintain silencing [12,13,14,22]. Here, we provide evidence that KRAB targeting alone might be sufficient to induce longer-term gene repression for certain genomic loci. Although KRAB proteins do not have catalytic activity themselves, they are considered strong indirect inducers of heterochromatin. In addition to the increase of H3K9me3 associated with the targeted region after ZF-KRAB expression, we also observed an increase of H3K27me3, which was in line for example with the observation that targeting KRAB to enhancers resulted in H3K27me3 at its interacting promoter [49]. This effect might relinquish the need for DNA methylation to achieve stability of KRAB-induced repression.

Despite the efficient DNA methylation induced by M.SssI, *PLOD2* repression was less pronounced (50% in fibroblasts; 75% in cancer cells) than SKD-induced repression (99% in both fibroblasts and cancer cell). Interestingly, in low-proliferating fibroblasts, but not in cancer cells, M.SssI induced repressive histone marks, in an almost similar pattern as SKD. However, the absolute level of these epigenetic modifications was lower for M.SssI, which was in line with the lower efficiency of M.SssI-induced transcriptional repression of *PLOD2*. Furthermore, targeting M.SssI did not repress unstimulated *PLOD2* expression, whilst both SKD fusions repressed *PLOD2* expression also in unstimulated conditions. This difference in effect might offer an advantage to targeted DNA methylation as an innovative clinical anti-fibrosis approach, although as for all epigenetic editing approaches, the specificity of the induced effects needs to be considered. As M.SssI is a highly active enzyme that generates broad methylation patterns [34], we included less active variants (e.g., C141S which has 1% activity of wild type M.SssI). Indeed, we could show induced methylation of only one CpG in the region where the C141S effector domain was predicted to be targeted, as was also observed by us previously using a different DNA binding platform [32]. However, methylation of this single CpG had no profound effect on chromatin modifications and transcriptional activity. When targeting Q147L (which has 10% activity of wildtype M.SssI enzyme) fused to dCas9 [50], we could observe downregulation of *PLOD2* expression. Downregulation was not observed with targeting the catalytically inactive mutant E186A, indicating that the repressive effect is not due to steric hindrance by dCas9 fusions.

Altogether, our results could imply that to achieve sustained repression of the *PLOD2* gene using the M.SssI enzyme, a threshold amount of DNA methylation has to be written and/or CpGs critical for transcription initiation need to be methylated. For *PLOD2*, a targeted methylation of 48.7% by ZF8 (Figure 4C), was not sufficient for significant repression after 10 days of TGFβ1 treatment in HDFs (Figure 2G), whilst a methylation of 63.4% by ZF7 did repress *PLOD2* in a significant manner. KRAB targeting alone did not result in increased methylation. This indicates that the KRAB-induced repressive effect for *PLOD2* is achieved through different (epigenetic) mechanisms, like the observed increase in H3K9me3 and H3K27me3, allowing innovative approaches to design small, single-component repressors to interfere with fibrosis.

## 4. Materials and Methods

### 4.1. Cell Culture and Stimulation

Human skin fibroblasts (adult donor) were obtained from American Type Culture Collection

(ATCC, Manassas, VA, USA) (CCD-1093SK) and cultured for up to 12 passages in EMEM (Lonza, Basel, Switzerland) supplemented with 10% heat inactivated fetal bovine serum (FBS) (Thermo Fisher Scientific, Waltham, MA, USA), penicillin/streptomycin (Lonza) and l-glutamine (Lonza). Fibroblasts isolated from the palmar fascia of a Dupuytren’s patient (according to Declaration of Helsinki principles) were a kind gift from Prof. Dr. P.M.N. Werker (University Medical Center Groningen, the Netherlands), and were cultured in DMEM (Lonza) supplemented with 10% heat-inactivated FBS, penicillin/streptomycin (Lonza) and l-glutamine (Lonza) for up to 6 passages. MDA-MB-231 breast cancer cells and Human embryonic kidney cells (HEK293T) were obtained from ATCC (CRM-HTB-26 and CRL-3216, respectively), and cultured in DMEM supplemented with 10% FBS, penicillin/streptomycin and l-glutamine. For experimental conditions the day after seeding, skin fibroblasts were serum-starved by a complete medium with 0,5% FBS (Thermo Fisher Scientific), 18 h prior to TGFβ1 stimulation. Recombinant human TGFβ1 (PeproTech, Rocky Hill, NJ, USA) was dissolved in 10 mM citric acid (Sigma Aldrich, St. Louis, MO, USA) pH 3.0 and diluted 20 fold with PBS supplemented with 0.1% bovine serum albumin (BSA) (Sigma Aldrich) to reach a concentration of 5 μg/mL. During all experimental procedures, the medium containing TGFβ1 (10 ng/mL) or an equal amount of vehicle control was refreshed daily.

### 4.2. Zinc Finger Design and Cloning

For targeting the *PLOD2* promoter by ZF technology, eight target regions, designated ZF1 t/m ZF8, were selected based on proximity to the TSS (RefSeq annotation) and on high-affinity predictions with the help of www.zincfingertools.org. DNA encoding the eight modular six-finger ZFs (Supplementary Figure S1) were synthesized (Bio Basic, Markham, ON, Canada) and subsequently cloned in the retroviral vector pMX-IRES-GFP either without effector domain (NoED) or containing the gene activator VP64, the transcriptional repressor SKD, or the DNA methyltransferase M.SssI. The latter effector domain was generated by PCR (Phusion Hot Start II High-Fidelity DNA polymerase, Thermo Fisher Scientific) on a previously described M.SssI carrying plasmid [33] using construct-specific PCR primers flanked with cloning restriction sites and ligated into the pMX-IRES-GFP with T4 ligase (Thermo Fisher Scientific). The less active derivatives of M.SssI (C141S and Q147L) and the inactive (double) mutant (E186A or Y137F+C141S) were acquired previously by site-directed mutagenesis [33]. ZF7 and ZF8 constructs were subcloned in the Retro-X TET-ON advanced doxycycline-inducible expression system (Takara Bio, Otsu, Japan).

### 4.3. Constructing dCas9 Fusions and Guide RNA Expression Plasmids

The plasmids pMLM3705 encoding the fusion protein dCas9-VP64 (Addgene, Watertown, MA, USA: plasmid #47754) and pMLM3636 used to express a single-chain guide RNAs (Addgene: plasmid #43860) were kind gifts from Keith Joung. Plasmids encoding single guide RNAs targeting *PLOD2* were generated by cloning 20 bp double-stranded oligonucleotides (see Supplementary Table S3) into BsmBI-digested pMLM3636.

The plasmids pM-dCas9-MSssI(Q147L) and pM-dCas9-MSssI(E186A), which transiently express the dCas9-MSssI(Q147L) or the dCas9-MSssI(E186A) fusion proteins in mammalian cells, were constructed by first cloning the M.SssI(C141S) allele into pdCas9-NED [51] (Addgene: plasmid #109358). The Q147L and E186A variants were created by fragment replacement using previously described M.SssI mutants [50]. The plasmids pHAGE EF1α dCas9-MSssI(Q147L) and pHAGE EF1α dCas9-MSssI(E186A) were used to stably integrate these transgenes into the genome of mammalian cells. The plasmids were constructed by cutting out the M.SssI(Q147L) and M.SssI(E186A) genes with SgsI(AscI) and PacI restriction enzymes (both; Thermo Fisher Scientific) and cloning them in the pHAGE EF1α dCas9-NED (Addgene: plasmid #109369) vector between the AsiSI and MluI sites as described before [52].

### 4.4. Viral Infections and Generating Stable Cells

For retroviral transduction of both types of fibroblasts with pMX-IRES-GFP, HEK293T cells seeded in 10cm dishes were transfected with 7.5 µg pMX-IRES-GFP together with 2.5 µg pMDg and 5 µg pMDg/p. The virus-containing supernatant of the HEK293T cells was harvested 48 h and 72 h after transfection, supplemented with 5% FBS and 6 μg/mL Polybrene (Sigma Aldrich) and centrifuged to remove cell debris. Host cells were seeded at 100,000 cells per 6-well and transduced with freshly made viral supernatant during two subsequent days. Three days after the last transduction, the host cells were either harvested or treated further with TGFβ1. To obtain stable inducible RetroX-TET-ON double transfectants, low passage number of skin fibroblasts or MDA-MB-231 cells were transduced following the same procedures as for pMX-IRES-GFP: although this time with 2.5 µg pRetroX-TET-ON, 5 µg pRetroX-Tight-Puro, 2.5 µg pMDg and 5 µg pMDg/p, and an extra virus filtration step. Heterogeneous populations of stable cells from the skin fibroblasts were obtained after selection with G418 sulfate (InvivoGen, San Diego, CA, USA) (600 μg/mL) and puromycin (InvivoGen) (1 μg/mL) for 7 days. In all cases to express the inducible ZF-fusion proteins in the stable cells, doxycycline (Takara Bio) (500 ng/mL) was supplemented to the culture medium for a total of 2 days, after which cells where harvested or sub-cultured, where MDA-MB-231 cells were split with 4–5 days interval.

The creation of the CRISPR-dCas9 expressing HEK293T stable cell lines has been described elsewhere [52]. Briefly, lentiviral pHAGE-EF1α constructs, encoding the dCas9-EDs were co-transfected with the second-generation packaging plasmids pCMVΔR8.91 and pCMV-VSV-G on day one into HEK293T cells using PEI transfection reagents (Polysciences Inc, Warrington, PA, USA) to produce lentiviral particles. The supernatant of HEH293T cells containing virus was harvested at 48 and 72 h after transfection. Host cells (in this case also HEK293T cells) were seeded in six-well plates and transduced on two consecutive days (day three and four) with 1.5 mL of the viral supernatant, supplemented with 8 µg/mL polybrene (Sigma Aldrich). The transduced cells were selected on day seven in 8 µg/mL puromycin-supplemented medium for four days and subsequently cultured in 1 µg/mL puromycin-supplemented medium.

### 4.5. Transient Transfection of Cells in CRISPR Experiments

Cells were transfected at 70% confluency in a six-well culture plate, using a total of 1 μg of DNA (500 ng sgRNAs and 500 ng dCas9-ED for wildtype cells and 1 μg sgRNAs for cells stably engineered to constitutively express the dCas fusion protein) using PEI in a 4:1 ratio. Forty-eight hours after transfection, 75% of the cells were harvested to assess their short-term effect on gene expression, and 25% were subcultured to assess long-term effects at 12 days. All transient transfection experiments were performed as biological triplicates.

### 4.6. DNA Methylation Analysis

Genomic DNA was isolated with phenol/chloroform extraction, treated with RNase and Proteinase K, and bisulfite converted (EZ DNA Methylation Gold kit; Zymo Research, Irvine, CA, USA). For bisulfite sequencing of *PLOD2* fragments, bisulfite converted DNA was amplified by PCR (Supplementary Table S1), ligated into a pCR2.1 TOPO vector with TA overhangs (Thermo Fisher Scientific) and transformed into TOP10 competent cells. Individual colonies were selected based on blue/white screening and the resulting plasmids were Sanger sequenced (BaseClear B.V., Leiden, the Netherlands) with a standard forward M13 sequencing primer. For pyrosequencing, bisulfite converted DNA was amplified by PCR using primers for two different *PLOD2* regions (Supplementary Table S1). Biotinylated PCR products were generated according to the PyroMark PCR kit manual (Qiagen, Venlo, the Netherlands). The samples were mixed with sequencing primers (Supplementary Table S1) and handled further according to the PyroMark Q24 (Qiagen) instructions. Quantitative DNA methylation levels were determined with the PyroMark software (Qiagen).

### 4.7. RNA Isolation and Quantitative RT-PCR

Total RNA was isolated (RNeasy Plus mini kit; Qiagen) or with TRIzol reagent (Thermo Fisher Scientific), quantified (NanoDrop; Thermo Fisher Scientific) and reverse transcribed into cDNA using random hexamer primers (RevertAid; Thermo Fisher Scientific). A mix of 10ng cDNA, primers (300nM) (Supplementary Table S1) and SYBR^®^ Green (Roche, Basel, Switzerland) was used for quantitative PCR analysis (ViiA7 platform and software; Thermo Fisher Scientific). Gene expression values of biological triplicates were either normalized to *GAPDH* or *YWHAZ* values using the standard ΔΔCt method. Fold expression levels were calculated from three independent experimental replicates.

### 4.8. Chromatin Immunoprecipitation

The chIP on fibroblasts and MDA-MB-231 cells was performed as described in [26], with ChIP-grade antibodies listed in Supplementary Table S2. Recovered ChIP DNA fragments were quantified with quantitative real-time PCR on the ViiA7 platform with primers (300nM) and probe (200nM) targeting genomic regions of *PLOD2* (Supplementary Table S1). The resulting qChIP data was calculated and represented as percent of input. 

### 4.9. Western Blotting and Immunocytochemistry

Proteins extracted from cells with RIPA buffer (Thermo Fisher Scientific), were fractionated by SDS-PAGE and transferred to nitrocellulose membranes. The membranes were blocked in TBS-T containing 5% skimmed milk powder, and incubated for 2 h at room temperature with primary antibodies against PLOD2 (R&D Systems, Minneapolis, MN, USA) or HA-tag (Abcam, Cambridge, UK). YWHAZ (Abcam) served as a loading control. Afterwards, the blots were incubated with secondary antibodies (goat-anti-rabbit-HRP or rabbit-anti-mouse-HRP (DAKO, Glostrup, Denmark)) for 1 h at room temperature, and chemi-luminescence was detected with ECL (Thermo Fisher Scientific). 

For immunocytochemistry, cells cultured in 24 wells plates or chamber slides (Thermo Fisher Scientific) were fixed with acetone/methanol (1:1 ratio) for 10 min at −20 °C. The cells were rehydrated with PBS for 10 min and blocked in 10% donkey serum (Abcam). Antibodies against PLOD2 (Sigma Aldrich) or Ki67 (Abcam) were incubated on fixed chamber slides for 1 h at room temperature. After washing with PBS, the slides were incubated for 30 min at room temperature with donkey anti-mouse A555-Cy3 (Thermo Fisher Scientific) (for PLOD2) or donkey anti-mouse A488-FITC (Thermo Fisher Scientific), (for Ki67) in DAPI (Thermo Fisher Scientific), with 2% BSA. Afterward, the slides were washed, mounted in Citi Fluor (EMS, Hatfield, PA, USA) and analyzed with the TissueFAXS (TissueGnostics, Vienna, Austria) fluorescence imaging system. For quantitation of positive cells, total areas of the wells were scanned and intensities were adjusted to the corresponding serotype control and calculated per nuclei (by DAPI; Thermo Fisher Scientific) with TissueQuest software (TissueGnostics). 

### 4.10. Statistics

Statistical tests were performed using Graphpad Prism 7 software (GraphPad, San Diego, CA, USA). Comparison between target conditions, and controls were investigated with an unpaired two-tailed Student’s *t*-test or one-way ANOVA, depending on the number of conditions. Differences were considered statistically significant when the *p*-value was * *p* < 0.05, ** *p* < 0.01, *** *p* < 0.001. All data are presented as the mean ±SEM of three biological replicates, unless stated differently. Pyrosequencing data were analyzed using a two-way ANOVA. 

## 5. Conclusions

In conclusion, our study provides an example of effective gene repression by KRAB-induced heterochromatin without targeted DNA methylation, which is resilient to continuous TGFβ1 transcriptional activation. This strategy of targeting *PLOD2* can be used to further develop epigenetic targeting approaches to prevent f.e. tissue fibrosis. As fibrosis still represents an unmet medical need, our approach together with the clinical progress of delivering gene-editing tools [3], might open a novel avenue towards personalized treatment options. 

## Supplementary Materials

Supplementary materials can be found at https://www.mdpi.com/1422-0067/21/10/3634/s1.

## Abbreviations

KRAB	Krüppel-associated box
SKD	Super KRAB Domain
dCas9	Deactivated Cas9
sgRNA	Single guide RNA
PLOD2	Procollagen-Lysine, 2-Oxoglutarate 5-Dioxygenase 2
ZF	Zinc finger
LH2	Lysyl hydroxylase 2
KAP-1	KRAB-associated protein 1
HP1	Heterochromatin Protein 1
CRISPR	Clustered regularly interspaced short palindromic repeats
M.SssI	CpG Methyltransferase
HEK293T	Human embryonic kidney cells
TALEN	Transcription activator-like effectors
ED	Effector domain
TGFβ1	Transforming growth factor beta-1
TSS	Transcription start site
HDFs	Human dermal fibroblasts
mRNA	Messenger RNA
DNA	Deoxyribonucleic acid
RNA	Ribonucleic acid
NoED	No effector domain
EV	Empty vector
CpGi	CG island
MTase	Methyltransferase
Dnmt	DNA methyltransferase
iPSCs	Induced pluripotent stem cells
FBS	Fetal bovine serum
DMEM	Dulbecco’s Modified Eagle Medium
BSA	Bovine serum albumin
PCR	Polymerase chain reaction
cDNA	complementary DNA
GAPDH	Glyceraldehyde 3-phosphate dehydrogenase
YWHAZ	14-3-3 protein zeta/delta (14-3-3ζ)
ChIP	Chromatin immunoprecipitation
RIPA	Radioimmunoprecipitation assay
SDS-PAGE	Sodium dodecyl sulfate-polyacrylamide gel electrophoresis
TBS-T	Tris-buffered saline and Tween 20
PBS	Phosphate-Buffered Saline
DAPI	4′,6-diamidino-2-phenylindole
ANOVA	Analysis of variance
