# Supplementary material for: KRAB-Induced Heterochromatin Effectively Silences PLOD2 Gene Expression in Somatic Cells and Is Resilient to TGFβ1 Activation
Source: Int J Mol Sci. 2020 May 21;21(10):3634. doi: 10.3390/ijms21103634 (PMC7279273; doi:10.3390/ijms21103634)
Supplement: Supplementary file 1 [file ijms-21-03634-s001.pdf]

Supplementary Information

KRAB-induced heterochromatin effectively silences *PLOD2* gene expression in somatic cells and is resilient to TGFβ1 activation

Rutger A. F. Gjaltema[1,2#], Désirée Goubert [1#], Christian Huisman[1], Consuelo del Pilar García Tobilla[1], Mihály Koncz[3,4], Pytrick G. Jellema[1,2], Dandan Wu[1], Uilke Brouwer [1,2], Antal Kiss[3], Pernette J. Verschure[5], Ruud A. Bank[2] and Marianne G. Rots[1*]

SUPPLEMENTAL MATERIALS

MCF7 cells were engineered to contain TET ON ZF-ED transgenes, as described for the MDA-MB-231 cells, or to constitutively express dCas9-fusion proteins as described for HEK293 cells. MDA-MB-231 cells, engineered to contain TET ON ZF-ED transgenes, cultured in DMEM and treated with doxycycline as described, were frozen after 20 days subculturing in 10%FBS complete medium after doxycycline removal (Figure 5, day 20). HDF cells, engineered to contain TET-ON ZF-ED transgenes and cultured in EMEM as described, were frozen (ca passage 8) before doxycycline treatment was performed. Upon thawing from liquid nitrogen, cells were either subcultured in culture medium supplemented with 10% regular FBS or in culture medium supplemented with 10% charcoal stripped FBS (Sigma-Aldrich). For HDF cells, FBS was heat inactivated as described before. MDA-MB-231 cells were seeded at a concentration of 150,000 cells per well and HDF cells were seeded at a concentration of 100,000 cells per well. Cells were collected at different time points as indicated, or seeded in a 6-well plate for further experiments. Depending on the research question, cells were treated with +/- doxycycline and +/- TGFβ1 as described (at day 25 for the transgenic MDA-MB-231 cells, at day 10 for HDF cells engineered to express dCas9-SKD and at day 21 for HDF cells engineered to express dCas9-MSssI). Expression of the ZF-EDs and *PLOD2* was measured with quantitative RT-PCR analysis, as described in the main manuscript.

SUPPLEMENTAL RESULTS

Leakiness of TET ON system explored

Although a degree of leakiness is often observed for the TET ON system, many reports have been published to demonstrate the transient nature of KRAB-induced gene repression in somatic cells (Groner et al., 2012; Amabile et al., 2016), also using TET ON transgenic cells (Rivenbark et al., 2012) without obvious leaky effects (Stolzenburg et al., 2012). However, since in our experimental system, *PLOD2* repression was apparent with and without doxycycline induction (Figure 7B, Supplemental Figure 7A), we decided to test whether trace amounts of doxycycline potentially present in the culture medium had any effect on the expression of ZF fusions. To this end, we thawed MDA-MB-231 cells stably expressing ZF7-SKD, ZF7-M.SssI, EV or ZF7-NoED

(Figure 7A, day 20) and cultured these cells for an additional 25 days either in 10% regular FBS medium or in 10% certified charcoal stripped medium (csFBS) which is depleted of lipophilic agents like doxycycline. After the total of 45 days subculturing after the first doxyxyxline treatment, a second doxyxycline treatment could still induce expression of the ZF-fusions (Supplemental Figure 7B). For all in between time points, the expression of ZF-EDs was not different in csFBS versus regular FBS (Supplementary Figure S8A). The similar expression levels in the two media did not support the notion that the observed leaky expression was caused by traces of doxycycline in regular FBS.

For either conditions, and at all time points, the repression of *PLOD2* by ZF-SKD was very effective, leading to a complete silencing of *PLOD2*, whilst ZF-M.SssI was able to repress *PLOD2* expression for 70%, independently of the growth conditions (Supplementary Figure S8B). Intriguingly, the leaky background expression of ZF-SKD was, at least on the mRNA level, about 10-fold higher than the background expression of ZF-M.SssI, which may explain the more effective repression by SKD. However, as each cell only has two *PLOD2* alleles, theoretically only two ZF-protein molecules can bind at any given time point. Since ZF-fusion proteins likely are present in excess after longer time points of culturing, it seems that the induced DNA methylation just cannot further repress the *PLOD2* expression, but this point requires more in depth investigation.

As we also saw doxycycline-independent *PLOD2* silencing in the HDFs stably engineered to express ZF-SKD or ZF.M.SssI (Figure 2), transgenic HDFs (frozen before dox treatment) were thawed and cultured up to 4 passages (i.e. 21 days for ZF-SKD cells and 30 days for M.SssI cells) in 10% csFBS or 10% regular FBS. If traces of dox in the FBS would be responsible for leaky expression of ZF-fusions, the SKD-induced repression of *PLOD2* would be relieved over time in cells subcultured in the csFBS medium, demonstrating that SKD functions transiently. Yet, again, we observed no difference in the expression of ZF-EDs, nor in the repression of *PLOD2*, between the two media (Supplementary Figures S8C, S8D). Interestingly, the leaky background expression of ZF-SKD in HDFs is about 100-fold higher than the background expression of ZF-M.SssI (Supplementary Figure 8C), even though no ZF-SKD protein expression could be detected (Figure 2C). Surprisingly, the uninduced background levels of ZF-SKD were sufficient to completely silence unstimulated *PLOD2* expression, while no repression was observed for M.SssI (Supplementary Figure S8D). These data are in line with observations for HDFs under conditions of doxycycline induction (Figure 2): although SKD effectively silenced the unstimulated *PLOD2* expression, M.SssI failed to repress endogenous *PLOD2* expression.

It is important to note that the actual doxycycline induction experiments in HDF cells were performed in low serum culture (20-fold lower: 0.5% regular FBS). Under such conditions, transient induction of expression of the ZF-fusions was achieved (Figure 2B). To assess whether the leakiness of the TET ON system was prevented by culturing HDFs in 0.5% FBS, we set out to investigate the leakiness of ED expression after long-term culturing in 10% vs. 0.5% FBS, in the presence or absence of dox (Supplementary Figure S9). Expression levels of ZF-fusions and EDs were assessed 2 days after the doxycycline treatment had ended, at which time-point ZF-ED expression had already returned back to uninduced (no doxycycline) levels (See Figure 2B). The very high expression generally observed for SKD was lower in the cells grown on 0.5% FBS +/- TGFβ1 +/- Dox whilst the expression of M.SssI was comparable in HDF cells grown in 10% *vs.* 0.5%

FBS (Supplementary Figure S9A). Despite the lower expression in 0.5% FBS, ZF-SKD resulted in strong *PLOD2* repression, whereas, *PLOD2* repression in ZF-M.SssI expressing cells was again comparable to that of NoED (Supplementary Figure S9B). As M.SssI cells treated with TGFβ1 did not survive the treatment, we could not assess how TGFβ1 induced-*PLOD2* expression was affected by ZF-M.SssI. Taken together, the results obtained with the TET ON expression system do not allow to make conclusions on the sustainability of targeted SKD- or M.SssI-induced effects on *PLOD2* expression.

REFERENCES

Amabile A, Migliara A, Capasso P, Biffi M, Cittaro D, Naldini L, et al. Inheritable Silencing of Endogenous Genes by Hit-and-Run Targeted Epigenetic Editing. Cell. 2016;167(1):219-32.e14.

Groner AC, Tschopp P, Challet L, Dietrich JE, Verp S, Offner S, et al. The Kruppel-associated box repressor domain can induce reversible heterochromatization of a mouse locus in vivo. J Biol Chem. 2012;287(30):25361-9.

Rivenbark AG, Stolzenburg S, Beltran AS, et al. Epigenetic reprogramming of cancer cells via targeted DNA methylation. Epigenetics. 2012;7(4):350-60.

Stolzenburg S, Rots MG, Beltran AS, Rivenbark AG, Yuan X, Qian H, Strahl BD, Blancafort P. Targeted silencing of the oncogenic transcription factor SOX2 in breast cancer. Nucleic Acids Res. 2012;40(14):6725-40.

SUPPLEMENTAL FIGURES

Figure S1

ZF1
GGATCCGAGGCCCAGGCGGCCCTCGAGCCCGGGGAGAAGCCCTATGCTTGTCCGGAATGTGGTAAGTCCTTCAGTGAACGTTCTCACCTTCGTGAACACCAGCGTAC
CCACACGGGTGAAAAACCGTATAAATGCCCAGAGTGCGGCAAATCTTTTAGTGATCCAGGCGCCCTGGTGCGCCATCAACGCACTCATACTGGCGAGAAGCCATACA
AATGTCCAGAATGTGGCAAGTCTTTCAGTACCAGCCACAGCCTGACCGAACACCAACGTACTCACACCGGGGAGAAGCCCTATGCTTGTCCGGAATGTGGTAAGTCC
TTCAGTGATCCAGGCGCCCTGGTGCGCCACCAGCGTACCCACACGGGTGAAAAACCGTATAAATGCCCAGAGTGCGGCAAATCTTTTAGTCGCAGCGATAACCTGGT
GCGCCATCAACGCACTCATACTGGCGAGAAGCCATACAAATGTCCAGAATGTGGCAAGTCTTTCAGTGATCCAGGCCACCTGGTGCGCCACCAACGTACTCACACCG
GTAAAAAAACTAGTGGCCAGGCCGGCCGAAGATCTGAGGAG

ZF2
GGATCCGAGGCCCAGGCGGCCCTCGAGCCCGGGGAGAAGCCCTATGCTTGTCCGGAATGTGGTAAGTCCTTCAGTACCAGCGGCGAACTGGTGCGCCACCAGCGTAC
CCACACGGGTGAAAAACCGTATAAATGCCCAGAGTGCGGCAAATCTTTTAGTCAGAGCAGCAACCTGGTGCGCCATCAACGCACTCATACTGGCGAGAAGCCATACA
AATGTCCAGAATGTGGCAAGTCTTTCAGTCGCAGCGATGAACTGGTGCGCCACCAACGTACTCACACCGGGGAGAAGCCCTATGCTTGTCCGGAATGTGGTAAGTCC
TTCAGTACCAGCGGCGAACTGGTGCGCCACCAGCGTACCCACACGGGTGAAAAACCGTATAAATGCCCAGAGTGCGGCAAATCTTTTAGTACCAGCGGCGAACTGGT
GCGCCATCAACGCACTCATACTGGCGAGAAGCCATACAAATGTCCAGAATGTGGCAAGTCTTTCAGTCGCAGCGATAACCTGGTGCGCCACCAACGTACTCACACCG
GTAAAAAAACTAGTGGCCAGGCCGGCCGAAGATCTGAGGAG

ZF3
GGATCCGAGGCCCAGGCGGCCCTCGAGCCCGGGGAGAAGCCCTATGCTTGTCCGGAATGTGGTAAGTCCTTCAGTCGCAGCGATAACCTGGTGCGCCACCAGCGTAC
CCACACGGGTGAAAAACCGTATAAATGCCCAGAGTGCGGCAAATCTTTTAGTCAGAGCGGCGATCTGCGCCGCCATCAACGCACTCATACTGGCGAGAAGCCATACA
AATGTCCAGAATGTGGCAAGTCTTTCAGTAGCCCAGCCGATCTGACCCGCCACCAACGTACTCACACCGGGGAGAAGCCCTATGCTTGTCCGGAATGTGGTAAGTCC
TTCAGTCGCAGCGATAAACTGGTGCGCCACCAGCGTACCCACACGGGTGAAAAACCGTATAAATGCCCAGAGTGCGGCAAATCTTTTAGTACCAGCGGCGAACTGGT
GCGCCATCAACGCACTCATACTGGCGAGAAGCCATACAAATGTCCAGAATGTGGCAAGTCTTTCAGTCGCAGCGATAAACTGGTGCGCCACCAACGTACTCACACCG
GTAAAAAAACTAGTGGCCAGGCCGGCCGAAGATCTGAGGAG

ZF4
GGATCCGAGGCCCAGGCGGCCCTCGAGCCCGGGGAGAAGCCCTATGCTTGTCCGGAATGTGGTAAGTCCTTCAGTGATCCAGGCCACCTGGTGCGCCACCAGCGTAC
CCACACGGGTGAAAAACCGTATAAATGCCCAGAGTGCGGCAAATCTTTTAGTCGCAGCGATAACCTGGTGCGCCATCAACGCACTCATACTGGCGAGAAGCCATACA
AATGTCCAGAATGTGGCAAGTCTTTCAGTCGCAGCGATAAACTGACCGAACACCAACGTACTCACACCGGGGAGAAGCCCTATGCTTGTCCGGAATGTGGTAAGTCC
TTCAGTCGCAGCGATCACCTGACCACCCACCAGCGTACCCACACGGGTGAAAAACCGTATAAATGCCCAGAGTGCGGCAAATCTTTTAGTGATCCAGGCGCCCTGGT
GCGCCATCAACGCACTCATACTGGCGAGAAGCCATACAAATGTCCAGAATGTGGCAAGTCTTTCAGTCAGCGCGCCCACCTGGAACGCCACCAACGTACTCACACCG
GTAAAAAAACTAGTGGCCAGGCCGGCCGAAGATCTGAGGAG

ZF5
GGATCCGAGGCCCAGGCGGCCCTCGAGCCCGGGGAGAAGCCCTATGCTTGTCCGGAATGTGGTAAGTCCTTCAGTGATTGCCGCGATCTGGCCCGCCACCAGCGTAC
CCACACGGGTGAAAAACCGTATAAATGCCCAGAGTGCGGCAAATCTTTTAGTGATTGCCGCGATCTGGCCCGCCATCAACGCACTCATACTGGCGAGAAGCCATACA
AATGTCCAGAATGTGGCAAGTCTTTCAGTCAGCGCGCCCACCTGGAACGCCACCAACGTACTCACACCGGGGAGAAGCCCTATGCTTGTCCGGAATGTGGTAAGTCC
TTCAGTCAGCGCGCCCACCTGGAACGCCACCAGCGTACCCACACGGGTGAAAAACCGTATAAATGCCCAGAGTGCGGCAAATCTTTTAGTCAGCGCGCCCACCTGGA
ACGCCATCAACGCACTCATACTGGCGAGAAGCCATACAAATGTCCAGAATGTGGCAAGTCTTTCAGTCGCAGCGATAACCTGGTGCGCCACCAACGTACTCACACCG
GTAAAAAAACTAGTGGCCAGGCCGGCCGAAGATCTGAGGAG

ZF6
GGATCCGAGGCCCAGGCGGCCCTCGAGCCCGGGGAGAAGCCCTATGCTTGTCCGGAATGTGGTAAGTCCTTCAGTCGCAGCGATAACCTGGTGCGCCACCAGCGTAC
CCACACGGGTGAAAAACCGTATAAATGCCCAGAGTGCGGCAAATCTTTTAGTGATCCAGGCCACCTGGTGCGCCATCAACGCACTCATACTGGCGAGAAGCCATACA
AATGTCCAGAATGTGGCAAGTCTTTCAGTCGCAGCGATAACCTGGTGCGCCACCAACGTACTCACACCGGGGAGAAGCCCTATGCTTGTCCGGAATGTGGTAAGTCC
TTCAGTCGCGCCGATAACCTGACCGAACACCAGCGTACCCACACGGGTGAAAAACCGTATAAATGCCCAGAGTGCGGCAAATCTTTTAGTGATTGCCGCGATCTGGC
CCGCCATCAACGCACTCATACTGGCGAGAAGCCATACAAATGTCCAGAATGTGGCAAGTCTTTCAGTCGCAGCGATAAACTGGTGCGCCACCAACGTACTCACACCG
GTAAAAAAACTAGTGGCCAGGCCGGCCGAAGATCTGAGGAG

ZF7
GGATCCGAGGCCCAGGCGGCCCTCGAGCCCGGGGAGAAGCCCTATGCTTGTCCGGAATGTGGTAAGTCCTTCAGTGATCCAGGCCACCTGGTGCGCCACCAGCGTAC
CCACACGGGTGAAAAACCGTATAAATGCCCAGAGTGCGGCAAATCTTTTAGTCGCAGCGATAAACTGACCGAACATCAACGCACTCATACTGGCGAGAAGCCATACA
AATGTCCAGAATGTGGCAAGTCTTTCAGTGATTGCCGCGATCTGGCCCGCCACCAACGTACTCACACCGGGGAGAAGCCCTATGCTTGTCCGGAATGTGGTAAGTCC
TTCAGTGATCCAGGCCACCTGGTGCGCCACCAGCGTACCCACACGGGTGAAAAACCGTATAAATGCCCAGAGTGCGGCAAATCTTTTAGTCAGAGCGGCGATCTGCG
CCGCCATCAACGCACTCATACTGGCGAGAAGCCATACAAATGTCCAGAATGTGGCAAGTCTTTCAGTCAGAGCGGCGATCTGCGCCGCCACCAACGTACTCACACCG
GTAAAAAAACTAGTGGCCAGGCCGGCCGAAGATCTGAGGAG

ZF8
GGATCCGAGGCCCAGGCGGCCCTCGAGCCCGGGGAGAAGCCCTATGCTTGTCCGGAATGTGGTAAGTCCTTCAGTCAGGCCGGCCACCTGGCCAGCCACCAGCGTAC
CCACACGGGTGAAAAACCGTATAAATGCCCAGAGTGCGGCAAATCTTTTAGTGATTGCCGCGATCTGGCCCGCCATCAACGCACTCATACTGGCGAGAAGCCATACA
AATGTCCAGAATGTGGCAAGTCTTTCAGTGATCCAGGCCACCTGGTGCGCCACCAACGTACTCACACCGGGGAGAAGCCCTATGCTTGTCCGGAATGTGGTAAGTCC
TTCAGTCGCAGCGATGAACTGGTGCGCCACCAGCGTACCCACACGGGTGAAAAACCGTATAAATGCCCAGAGTGCGGCAAATCTTTTAGTGATTGCCGCGATCTGGC
CCGCCATCAACGCACTCATACTGGCGAGAAGCCATACAAATGTCCAGAATGTGGCAAGTCTTTCAGTCGCAGCGATAAACTGGTGCGCCACCAACGTACTCACACCG
GTAAAAAAACTAGTGGCCAGGCCGGCCGAAGATCTGAGGAG

Figure S1, related to figure 1. *PLOD2* ZF coding sequences.
Synthesized DNA coding for the eight engineered zinc finger proteins (ZF1-8) that target *PLOD2*. Depicted in red are the cloning sites and in blue the six individual fingers that make up the ZF.

Figure S2

A

ZF1 GGC GAG GTC CCA GTC AGC

ZF2 GAG GCT GCT GTG GAA GCT

ZF3 GGG GCT GGG ACA GCA GAG

ZF4 GGA GTC TGG CGG GAG GGC

ZF5 GAG GGA GGA GGA GCC GCC

ZF6 GGG GCC CAG GAG GGC GAG

ZF7 GCA GCA GGC GCC CGG GGC

ZF8 GGG GCC GTG GGC GCC TGA

B

```
-1300 ACTGGAGAAC CAAGGAAGCA AGAAGTCAAG TTCAAGGAGT TTGGCAATTG CAGGTTTGGC TTTCTGGAGA CAGATCTGGC CAAAACACCA GCCATTCCTA
-1200 GCAGAGTCCT TGGACTCAGA GGACTGATGC TGTTTGCTTT GTGCCTAGTT TTATGAAGCT GTACTTGCCA CACATGAAAG GCTGTTATTA AGGAATCCAT
-1100 CTTTTAGCTT TTTTTTTCCC CTTCAGACTA AATAAACCTT ATTTCATTCT TCTCACAGGT CATAGTTTCC ACTTCTTTAA TTATTTGTTA CTCTTCTCAG
-1000 AACTTTCTCC AGTTTTCCTC TTCTTTTAAA AAACTGAGAC CAAACCTTCA AAACAGAGTA ATAACTGGCT TAGTTCTGGG CACAGGAAAT GATTCTTTCT
 -900 AAAATGTCAC ATCCCAGGCA AGATTTCTTT GCAGGGTTTT CCACCTGTTT TTAATGCTAT CAAGATGAAA GCATTAAGTT CATGTTATCT TGATATGTTA
 -800 ATTTTAGACA AAACGTGATC ATAATGGAAC ACATTTTTCC ATAATTTTCT GTCTTAATTT CAAAGGCCCA GAGTTATAAC GGGTGTTGTC TCGATTCTGT
 -700 TTTTTTCTAA TTTCACGAAA ATTTAGGCAG GAGATTCCAG AATGGTAGCA GTTTGGTTCT CTTTCTCCTA TAAAATAGCT TATGTCATTA AATGTATGAG
 -600 GCAAAACTGT CAAGCTTTTT TCTTAATTGT GCTTTTGCTC CCACTCATTC CAACAAGTTA AACCCTTGGC TAGTTAAAAT GTGAGAACAG AAAAAAGGGA
 -500 ATTTTCTTAG ACATCAGTCA AAAGTGGAAG CAGTGGGGGC CAAGGCTCAG AGATCTCGAC CTCTCCGACC ACGTCTTTGG GAGTAATTTT CCATGCGGAC
 -400 TGGAGCACGA TCTTGTAAGC ACAGCCCCGG CATTACACAC GTTGGCTCCC GATTCCGCAG GATTTACAAG CCTCATTACT GGAAAGAGTC TAAGGCTCTC
 -300 TTGGCACAAG CGGCGGAGCA AGAAATGTTC ACTGGACAAT AAAACTTTCG GTTTACGGAG TAAAATAAAA TCACGGCCTT CCTTTTACAG GGTGATGAAC
 -200 CTGCCCCTGA ATTAATGAGG GGCGATCCAT TTCCATGGCT TAAGAGTCTG GCGAGGTCCC AGTCAGCCAA GATTGCCGCA GATTTGCAGT CACTTCAGAC
                                                              ZF1
 -100 TGGGTGGAGT ATCCAAGTCC ATAGAAGAGC AAATTCTCAC CCTTCGAGGC TGCTGTGGAA GCTACCGGGG CTGGGACAGC AGAGAGGAAC CCAGACGGGA
                                                       ZF2                    ZF3
    1 ACACCGCCCT CCCGCCAGAC TCCCGGCGGC TCCTCCTCCC TCTCCCAAAC CCACTCCCAA AGCTAAGTGC AGGCTTCCCC GTTCCAGCCA GAAGCGCTGC
                 ZF4             ZF5
  101 GTGAGCCTCC ACACGTAGCC GCAGGCAGCT CCTTAAATAG CGTCCGCGCT GAGCAAACAG TCCAGACGTG GGGCCCAGGA GGGCGAGCTG AGGCGACCGC
                                                                           ZF6
  201 ACCGGGCGCG CAGCGGCGGC GGGTCAGCCG GCGGCCAATA GCCAGGGCGC GGCCCGCCCC GTCGCCTCCC CTCGGGGAGC CTATAAGGCC TCCGCAGCGC
  301 CCCGGGCGCC TGCTGCTCCG TGCCTCCACC GACGACCTCA CTCAGCTGCG TTACGCGCCG CTCCGGCTGC CGGCCGCGCG CCTTGCCCGC CGGCTCCCGC
          ZF7
  401 CCGCAATCGG CGGCTCAGGG CGGACCCGGG TCTCTGCGTT CTCGCGAGAA GCGCGGCGCT GCGGGGCCGT GGGCGCCTGA GCCCGCGCGG CCCTCGAGGG
                                                                         ZF8
  501 CCGAATATGG GGGGATGCAC GGTGAAGCCT CAGCTGCTGC TCCTGGCGCT CGTCCTCCAC CCCTGGAATC CCTGTCTGGG TGCGGACTCG GAGAAGCCCT

ATATG = exon
CTGAC = ZF binding site
```

Figure S2, related to figure 1. Zinc Finger targeting sites.
(A) Recognition sites (18bps) of the eight Zinc Fingers designed to target *PLOD2*. **(B)** Promoter overview of *PLOD2* from -1300/+600 bps, with the ZF binding sites and CpGs (in red).

Figure S3

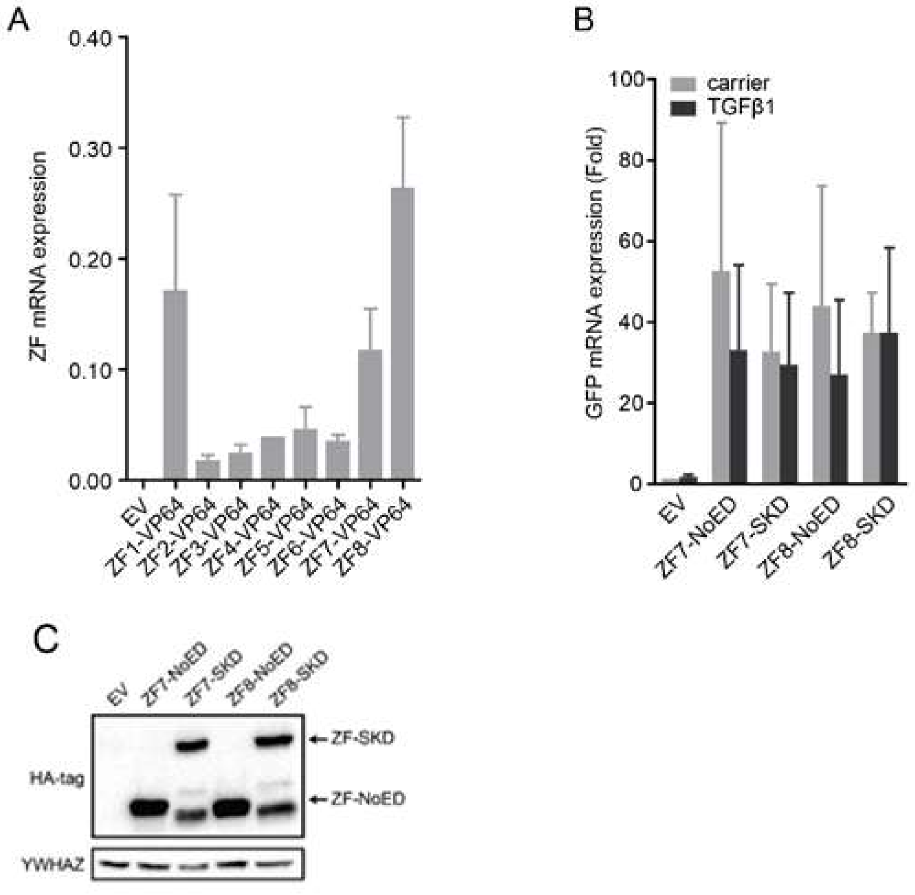

Figure S3, related to figure 1. ZF expression in fibroblasts.
(A) ZF-VP64 mRNA expression levels of HDFs transduced with retrovirus for the eight ZF-VP64 fusion proteins or EV control (mean ± SEM; n = 3). **(B)** GFP mRNA expression of HDFs transduced with retrovirus for ZF-7/8 alone (NoED) or in fusion with SKD treated with TGFβ1 for two days. GFP is expressed via an IRES of the ZF transcripts (mean ± SEM; n = 3). **(C)** Western blot of ZF expression in Hek293T cells 2 days after transfection with EV, ZF7 and ZF8 NoED and SKD fusion plasmids, stained against HA tag and YWHAZ as a loading control.

Figure S4

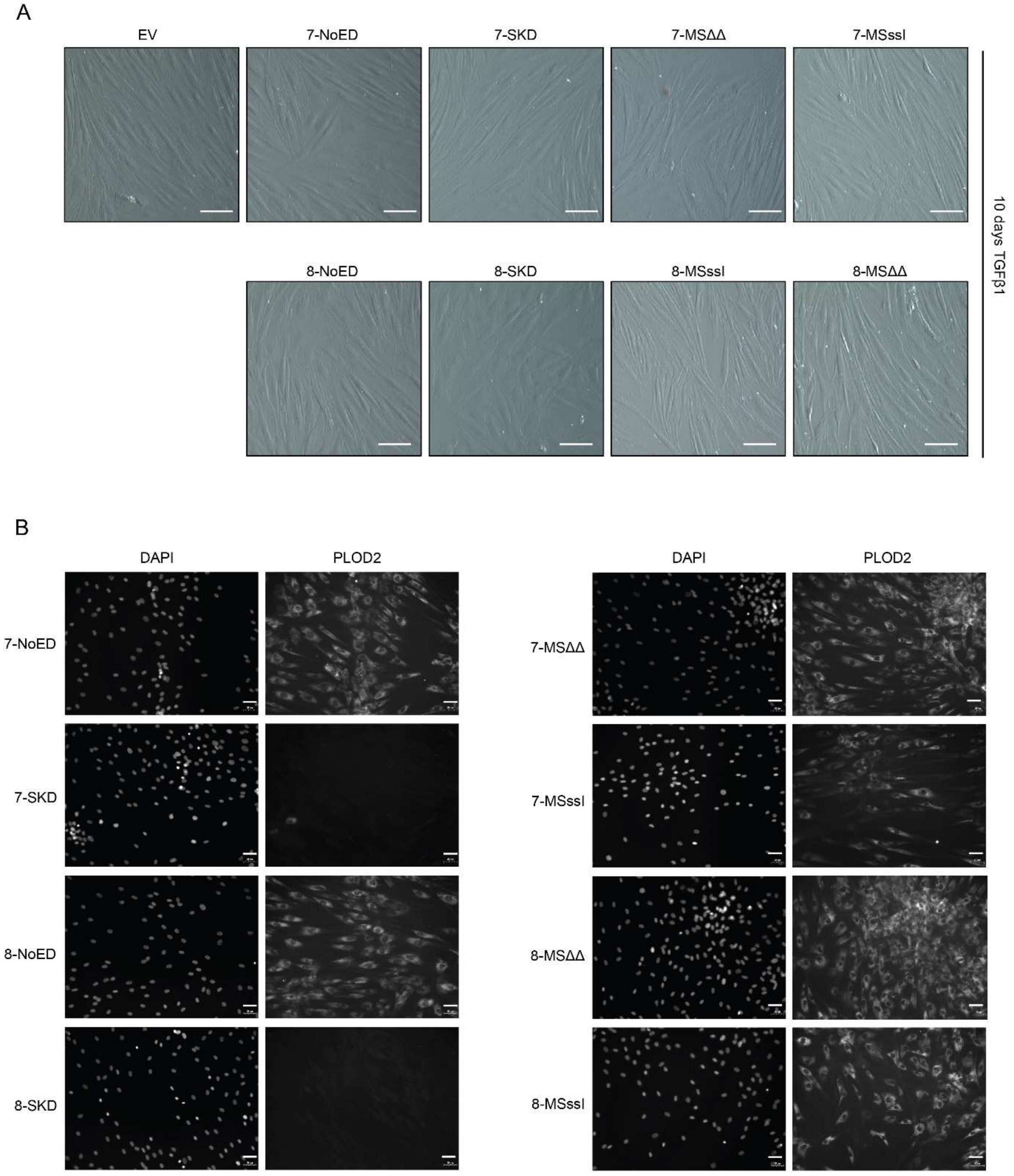

Figure S4, related to figure 2. PLOD2 expression and fibroblast morphology after 10 days of TGFβ1 stimulation. (A) Phase contrast images of doxycycline induced HDFs that were stimulated with TGFβ1 for 10 days. White bars represent 10µm. **(B)** Immunocytochemistry of PLOD2 and DAPI from doxycycline induced HDFs and subsequent TGFβ1 stimulation for 10 days. White bars represent 50µm.

Figure S5

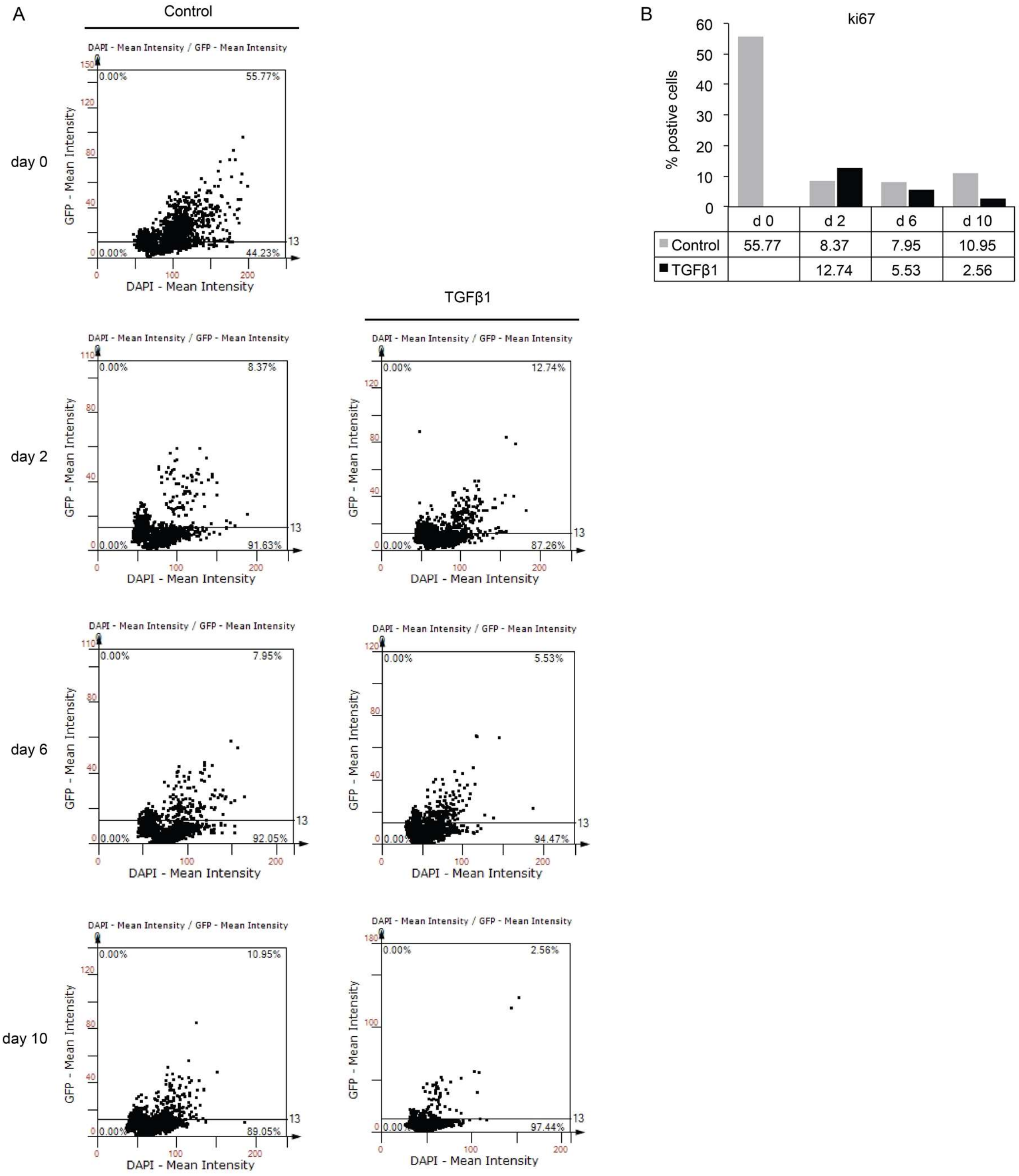

Figure S5, related to Figure 3. Culturing HDF in low serum conditions and TGFb1 stimulation reduces proliferations over time. (**A**) TissueFAX quantifications plots from Ki67 stainings of HDF cultured in 10% FBS (day 0) or when treated with TGFβ1 (in 0.5% FBS) or control (in 0.5% FBS) for 2, 6 or 10 days. Each dot represents one cell (**B**) Barplot showing data from **A**.

Figure S6

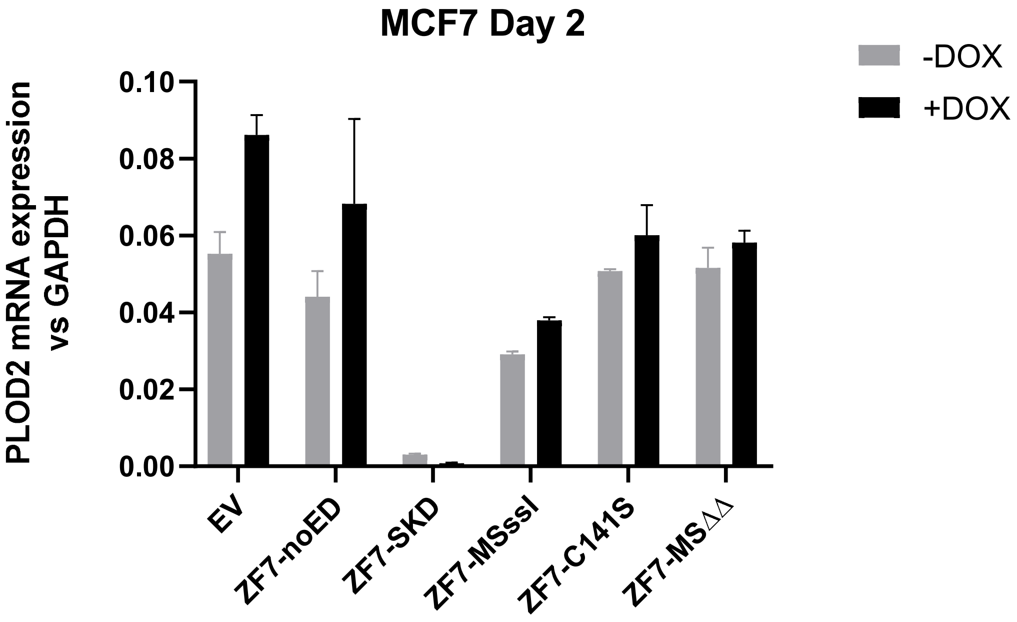

Figure S6, related to figure 7. Targeting SKD and M.SssI resulted in *PLOD2* repression in MCF-7 breast cancer cells engineered to express the indicated ZF-fusions. *PLOD2* mRNA expression levels in MCF-7 cells after 2 days with or without doxycycline treatment.

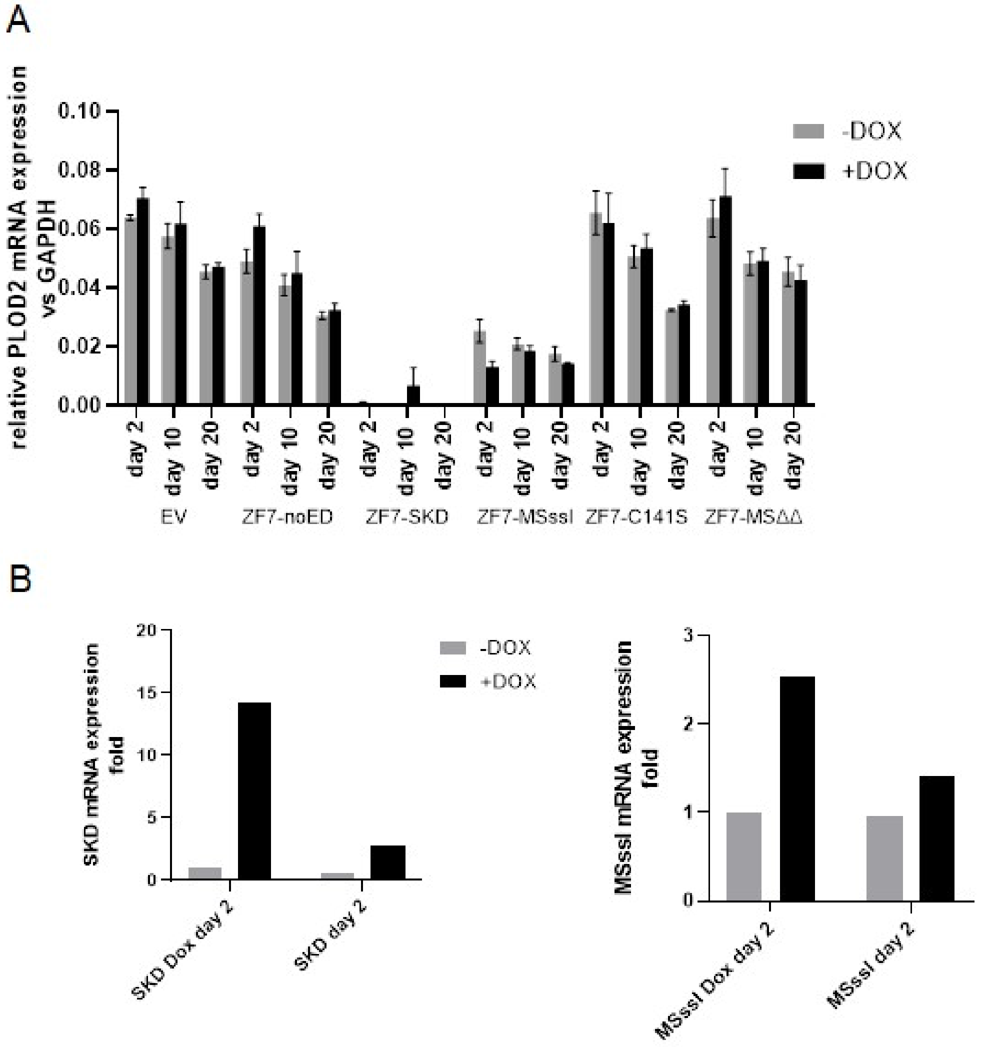

Figure S7. (**A: related to Figure 7**) RNA expression levels of *PLOD2* in transgenic MDA-MB-231 cells measured with or without two days doxycycline treatment followed by subculturing for 2, 10 or 20 days as indicated, (**B**) RNA expression levels of ZF-SKD or ZF-M.SssI fusions in thawed transgenic MDA-MB-231 cells measured directly after the treatment with doxycycline for two days (Dox day 2) or after 2 days of subculturing (day 2).

Figure S8

MDA.MB-231 cells

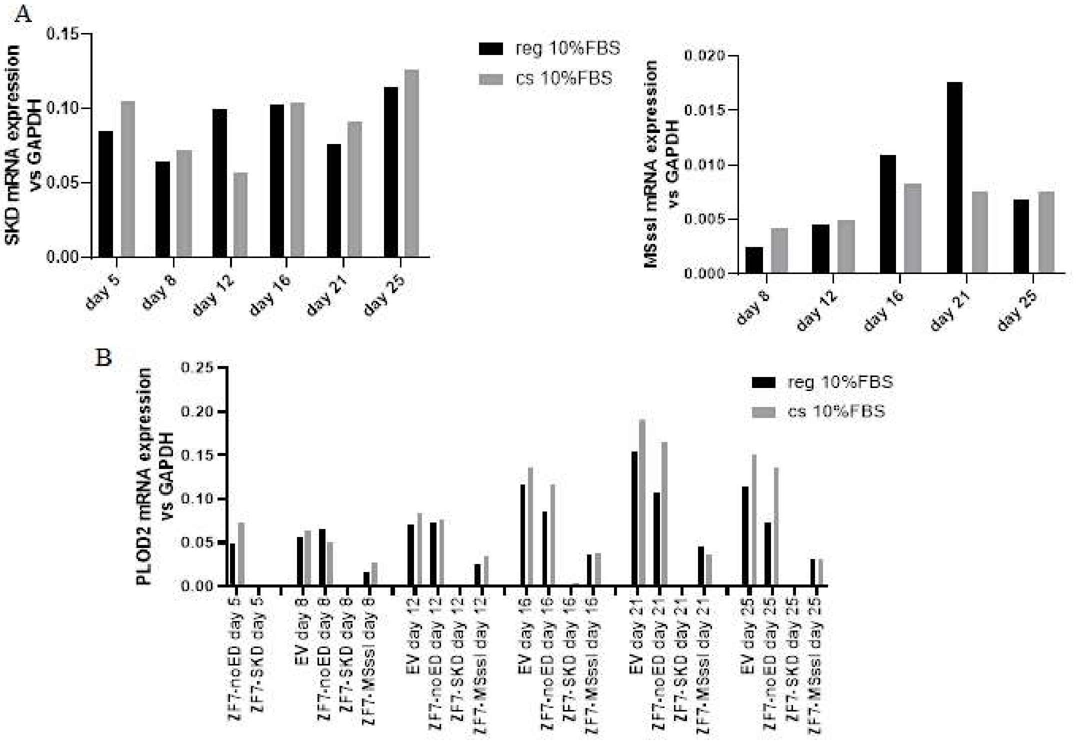

Human Dermal Fibroblasts

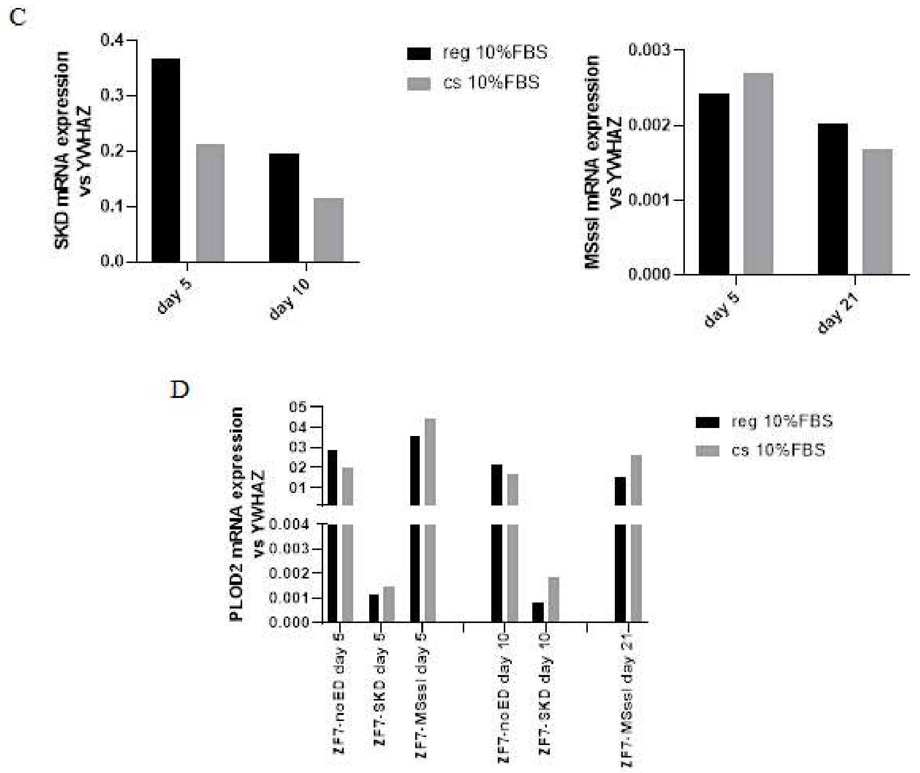

Figure S8 No difference in the expression of the ZF-EDs, or *PLOD2,* in cells grown in regular FBS *vs.* Charcoal Stripped (CS) medium. **(A)** ZF-SKD and ZF-M.SssI or **(B)** *PLOD2* expression in MDA-MB-231 cells (frozen at day 20 after dox treatment (Figure 7)) after culturing in regular vs. csFBS medium for the indicated period; **(C)** ZF-SKD and ZF-M.SssI or **(D)** *PLOD2* expression in transgenic HDFs (frozen before doxycycline treatment) after growing in regular vs. csFBS for the indicated period. Data are presented as -fold values relative to those of *GAPDH* (MDA) or *YWHAZ* (HDF).

Figure S9

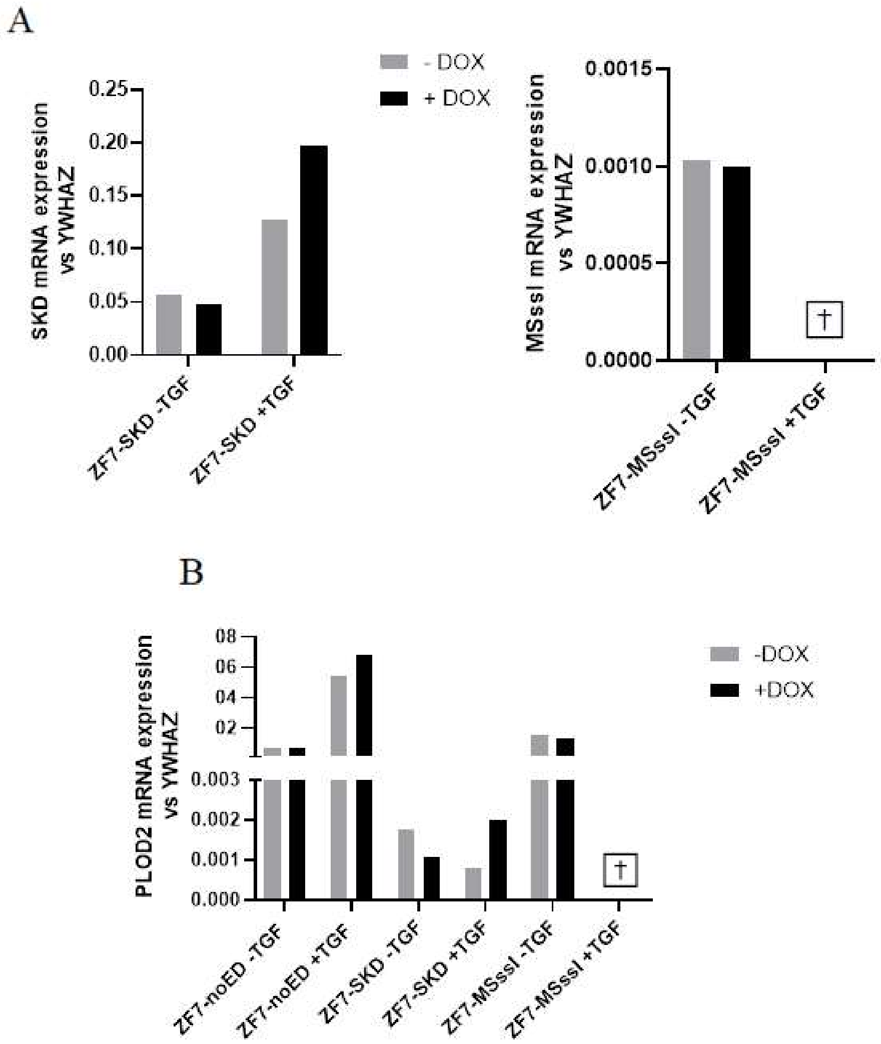

Figure S9. No clear difference in background expression of the ZF-ED in HDFs with or without TGFβ1 treatment. HDFs were grown in 0.5% FBS and first treated with or without Dox for two days, followed by +/- TGFβ1 treatment for 2 days. **(A)** ZF-SKD and ZF-M.SssI or **(B)** *PLOD2* expression presented as fold-values relative to those of *YWHAZ*. † = no data could be obtained as all cells died.

Figure S10

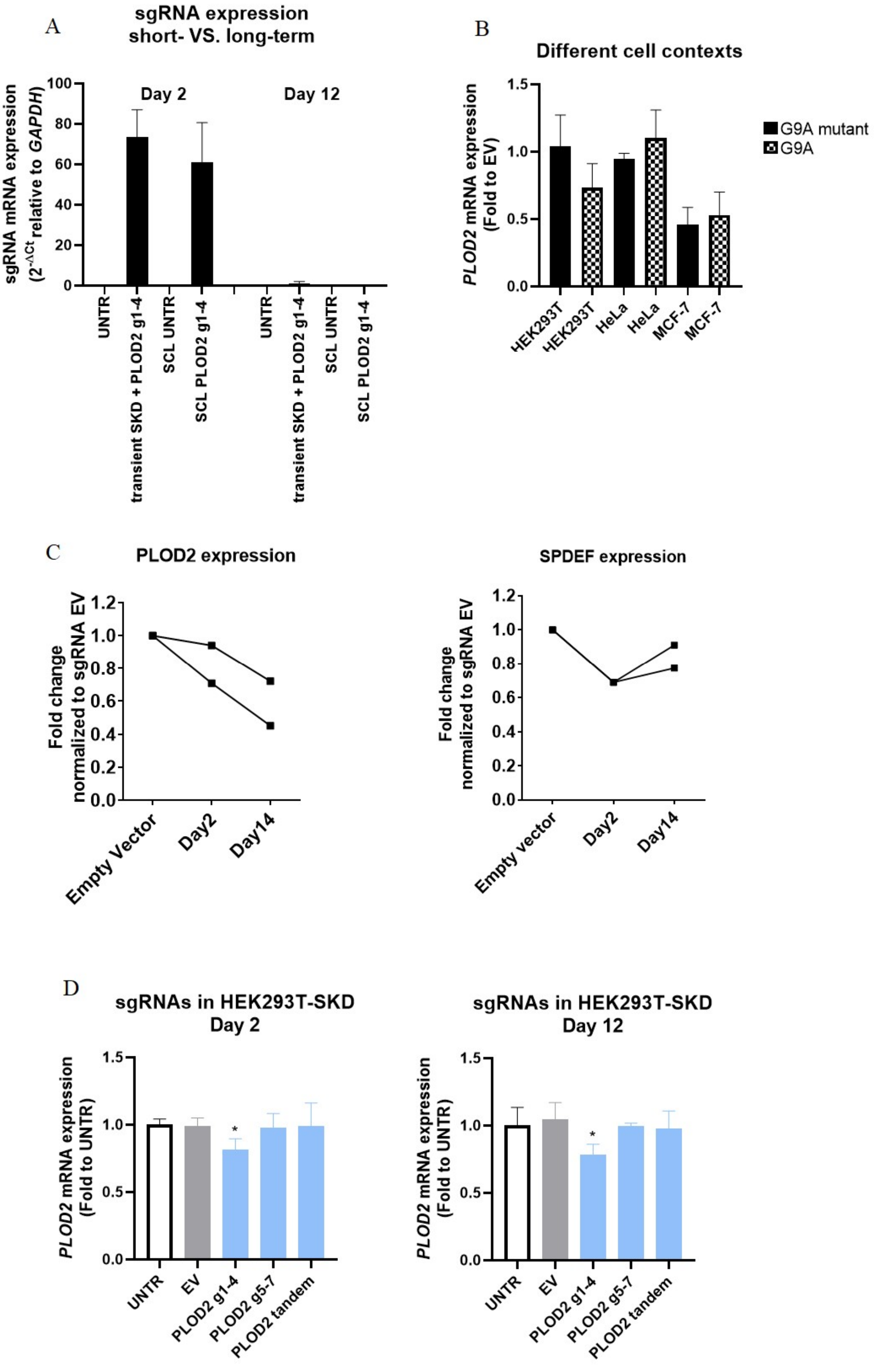
A
sgRNA expression
short- VS. long-term
sgRNA mRNA expression
(2-ΔCt relative to GAPDH)
Day 2
Day 12
UNTR
transient SKD + PLOD2 g1-4
SCL UNTR
SCL PLOD2 g1-4
B
Different cell contexts
PLOD2 mRNA expression
(Fold to EV)
G9A mutant
G9A
HEK293T
HeLa
MCF-7
C
PLOD2 expression
SPDEF expression
Fold change
normalized to sgRNA EV
Empty Vector
Empty vector
Day2
Day14
D
sgRNAs in HEK293T-SKD
Day 2
Day 12
PLOD2 mRNA expression
(Fold to UNTR)
UNTR
EV
PLOD2 g1-4
PLOD2 g5-7
PLOD2 tandem

Figure S10, related to figure 8. *PLOD2* expression modulation using the CRISPR-dCas9 platform. (A) sgRNA expression in HEK293T cells transiently transfected or HEK293T-SKD stable cells measured after 2 or 12 days of transfection. Expression is presented relative to *GAPDH.* **(B)** *PLOD2* mRNA expression levels after 2 days of different cell lines constitutively expressing G9A or G9Amutant, transiently transfected with sgPLOD2 1-4 (mean ± SEM; n = 3, unpaired two-way Student's t-test *=$P < 0.05$). **(C)** *PLOD2* and *SPDEF* mRNA expression levels, 2 and 14 days after transient transfection with sgRNAs in MCF-7 cells engineered to constitutively express dCas9-SKD cells, normalized to expression in cells transfected with empty vector (n=2). (**D**) Stable HEK293T-SKD cells transiently transfected with different groups of sgRNAs (mean ± SEM; n = 3, unpaired two-way Student's t-test *=P < 0.05)

Table S1. Primers used in this study for mRNA qPCR, bisulfite sequencing, pyrosequencing and qChIP.

Target	Forward 5′-3′	Reverse 5′-3′
qPCR *PLOD2*	GGGAGTTCATTGCACCAGTT	GAGGACGAAGAGAACGCTGT
qPCR *GAPDH*	CTGCCGTCTAGAAAAACCTG	GTCCAGGGGTCTTACTCCTT
qPCR *YWHAZ*	GATCCCCAATGCTTCACAAG	TGCTTGTTGTGACTGATCGAC
qPCR GFP	ACGTAAACGGCCACAAGTTC	AAGTCGTGCTGCTTCATGTG
qPCR ZF-VP64	AAGCGACGCATTGGATGAC	GGAACGTCGTACGGGTAGTTAATT
qPCR ZF-SKD	CAGATGTGATCCTCCGGTTG	TGCGTTTCTTTTTCGGAACT
qPCR ZF-M.SssI	CTGGAATTGGTGCTCAAAGAA	CAGGAACGTACCATTCAGCA
qPCR *SPEDF*	TGTCCGCCTTCTACCTCTCCTAC	CGATGTCCTTGAGCACTTCGC

Target	Forward 5′-3′	Reverse 5′-3′
Bisulfite seq *PLOD2*	TTAAAGTTAAGTGTAGGTTTTT	AAAACAACAACTAAAACTTC

Target	Forward 5′-3′	Reverse 5′-3′	Sequencing 5′-3′
Pyroseq *PLOD2* +349/+443	GTTTTTTTTAGGGGAGTTT ATAAGGT	[Btn]CTAAAACTTCACCCTACA TCCCCCCATAT	GTTTTTATAGAAGATTTTAT TTAGT
Pyroseq *PLOD2* -443/-372	AAGTAGTGGGGGTTAAGG	[Btn]CCAAAAAAACCTTAAACT CTTTCCAATAAT	GGGTTAAGGTTTAGAGA

Target	Forward 5′-3′	Reverse 5′-3′	Probe 5′-3′
ChIP *PLOD2* +326/+447	CACCGACGACCTCACTCA	TCGCGAGAACGCAGAGAC	[FAM]CTGCGTTACGCGCCG CTC [TAMRA]
ChIP *PLOD2* -796/-686	TTAGACAAAACGTGATCA TAATGGA	ATCTCCTGCCTAAATTTTCGTG	[FAM]TCAAAGGCCCAGAG TTATAACGGGTG[TAMRA]

Table S2. Antibodies used in this study for Western blotting, immunocytochemistry and ChIP.

Antibody	Supplier	Catalogue #
PLOD2	R&D system	MAB4445
PLOD2	Sigma Aldrich	SAB1400213
YWHAZ	Abcam	ab51129
HA-tag	Abcam	ab9110
H3ac	Millipore	06-599
H3K4me3	Millipore	07.473
H3K9me3	Abcam	ab8898
H3K27me3	Millipore	07-449
H3	Abcam	ab1791
Rabbit-anti-mouse-HRP	DAKO	P0260
Goat-anti-rabbit-HRP	DAKO	P0448

Table S3. Oligonucleotides designed to generate *PLOD2* targeting single-guide RNAs

guide RNA #	Oligo A 5′-3	Oligo B 5′-3
PLOD2:		
g1	ACACCGCTGTGGAAGCTACCGGGGCG	AAAACGCCCCGGTAGCTTCCACAGCG
g2	ACACCCCACTCCCAAAGCTAAGTGCG	AAAACGCACTTAGCTTTGGGAGTGGG
g3	ACACCGAGCCTCCACACGTAGCCGCG	AAAACGCGGCTACGTGTGGAGGCTCG
g4	ACACCTGAGCAAACAGTCCAGACGTG	AAAACACGTCTGGACTGTTTGCTCAG
g5	ACACCGCATGGCTTAAGAGTCTGGCGG	AAAACCGCCAGACTCTTAAGCCATGCG
g6	ACACCGATAAGGCCTCCGCAGCGCCCG	AAAACGGGCGCTGCGGAGGCCTTATCG
g7	ACACCGTCGCGAGAAGCGCGGCGCTGG	AAAACCAGCGCCGCGCTTCTCGCGACG
SPDEF:		
g1	ACACCGCATGGATCCCCCAGCAAGGG	AAAACCCTTGCTGGGGGATCCATGCG
g2	ACACCCCTCAGGTTGGGCCTTGCCAG	AAAACTGGCAAGGCCCAACCTGAGGG
g3	ACACCCTGGCCAACTCTTCATCTCGG	AAAACCGAGATGAAGAGTTGGCCAGG
